# The kinesin-14 family motor protein KIFC2 promotes prostate cancer progression by regulating p65

**DOI:** 10.1016/j.jbc.2023.105253

**Published:** 2023-09-14

**Authors:** Xinyu Liu, Yu Lin, Weibing Long, Renzheng Yi, Xiongfeng Zhang, Chaoqun Xie, Na Jin, Ziran Qiu, Xiaobing Liu

**Affiliations:** 1 Department of Urology, Loudi City Central Hospital, Loudi, Hunan, China; 2 Department of Urology, Shanghai Ninth People's Hospital, Shanghai Jiao Tong University School of Medicine, Shanghai, China; 3 Department of Surgical Oncology, Loudi City Central Hospital, Loudi, Hunan, China

**Keywords:** prostate cancer, KIFC2, tumor progression, chemotherapeutic resistance, NF-κB pathway

## Abstract

The kinesin-14 motor proteins play important roles in tumor development and drug resistance and have been reported as potential biomarkers or therapeutic targets for tumor treatment. However, kinesin family member C2 (KIFC2), one of the kinesin-14 motor family members, remains largely unknown in prostate cancer (PCa) progression. Here, we used the GEO and The Cancer Genome Atlas datasets, Western blotting, and immunohistochemistry analyses to detect KIFC2 expression in PCa tissues. Additionally, a series of *in vivo* and *in vitro* experiments were utilized to demonstrate the roles of KIFC2 in PCa cells. We found that KIFC2 was highly expressed and positively correlated with the clinicopathological characteristics in PCa. Functional experiments indicated that KIFC2 could promote PCa progression. Furthermore, we performed an analysis of the KEGG and GSEA databases, subcellular fractionation, and immunofluorescence to investigate the potential mechanisms of KIFC2 in PCa. We confirmed that KIFC2 could regulate the NF-κB pathway *via* mediating NF-κB p65 protein expression and nuclear translocation thereby promoting PCa progression and chemotherapeutic resistance. Together, our results suggest that KIFC2 is overexpressed in PCa. By regulating the NF-κB pathway, KIFC2 may play a crucial role in PCa.

As the one of the most common cancer types in man, treatment failure and disease progression of prostate cancer (PCa) continue to plague clinicians. At present, androgen-deprivation therapy is the first-line treatment for PCa patients (1). Although initially responsive, most PCa patients will progress to a castration-resistant state, which represents disease recurrence, metastasis, and antiandrogen treatment resistance (2, 3). However, the underlying mechanisms of metastasis and antiandrogen treatment resistance of PCa remain unclarified. Enzalutamide (Enza), a second-generation androgen receptor (AR) inhibitor *via* disrupting the translocation of the AR from the cytoplasm into the nucleus and impairing binding of the AR to the transcriptional complex, is approved by FDA to treat PCa patients with different stages of the disease, including castration-resistance prostate cancer (CRPC) (4, 5). Nevertheless, most PCa patients invariably develop resistance against Enza and die from hormone-refractory PCa, therefore become a major clinical challenge (6).

The kinesin-14 motor family member, kinesin family member C2 (KIFC2), is especially expressed in neurons and is important for organizing dendritic microtubules and to control dendrite development (7). In mammals, there are three kinesin-14 members, kinesin family member C1, KIFC2, and kinesin family member C3. It was recently reported that kinesin family member C1expression increases abnormally and contributes to multidrug resistance in various cancers, including PCa (8, 9, 10, 11). In addition, kinesin family member C3 was also shown to promotes malignant phenotypes and drug resistance (12, 13, 14). However, our knowledge of KIFC2’s function in PCa is still limited.

NF-κB is a family of transcription factors implicated in a variety of physiological and pathological processes (15). The key step of activation of the canonical NF-κB is phosphorylation-dependent activation of the IκB kinases complex (16). The most common form of NF-κB is the heterodimer of the p50 and p65 subunits. The p65 subunit have a transcriptional activation domain and is involved in cell proliferation, metastasis, angiogenesis, and chemotherapy resistance (17, 18). p65 activity is regulated at multiple levels. Therefore, only understanding the nuclear localization of p65 is not enough. Understanding the molecular mechanisms underlying the regulation of the subunits of the NF-κB complex and determining their functions will help elucidate the NF-κB pathway as a whole. Currently, studies on the regulation of p65 expression and nuclear translocation by KIFC2 remain scarce, and this study aims to investigate how KIFC2 mediates the PCa progression *via* regulating p65.

## Results

### Identification of KIFC2 related to Enza-resistant in PCa

**Figure 1:**
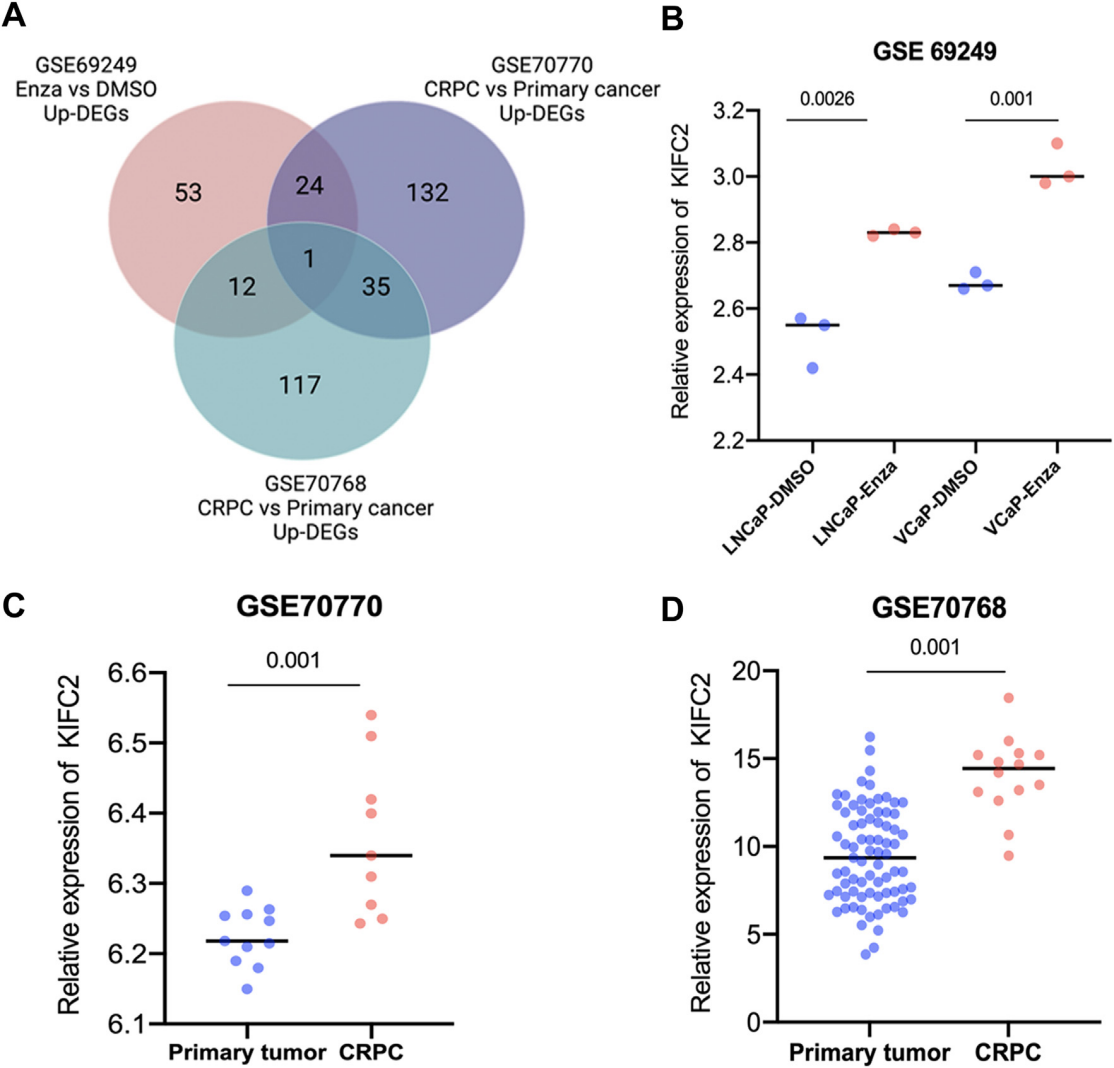
**KIFC2 expression is related to enzalutamide-resistance PCa and CRPC.***A*, the Venn diagram gives the overlapped genes of high expression related to enzalutamide-resistance and CRPC based on three GEO datasets. *B*, the expression of KIFC2 and that in comparison among enzalutamide-treated and DMSO-treated cells in GSE69249 dataset. *C* and *D*, KIFC2 expression of CRPC and primary tumor in the GSE70770 dataset and GSE70768 dataset. Data are mean ± SD (n = 3). CRPC, castration-resistance prostate cancer; DEGs, differentially expressed genes; Enza, enzalutamide; KIFC2, kinesin family member C2; PCa, prostate cancer.

To explore potential genes that may regulate PCa progression and Enza resistance, we analyzed three GEO datasets (GSE69249, GSE70770, and GSE70768) to identify Enza resistance– and CRPC-related genes. Cross-comparison of all clustered genes allowed us to identify KIFC2 that were significantly elevated in the Enza-resistance group and CRPC group (>2-fold change, *p* < 0.05; Fig. 1*A*). We found that KIFC2 level was higher in the Enza-treated cells than in the DMSO-treated cells in LNCaP and VCaP cell lines in GSE69249 (Fig. 1*B*) and that KIFC2 expression was higher in CRPC that in primary PCa (Fig. 1, *C* and *D*), which revealed a positive correlation between KIFC2 and PCa progression and Enza resistance.

### KIFC2 is elevated and correlated with unfavorable pathological characteristics in PCa

**Figure 2:**
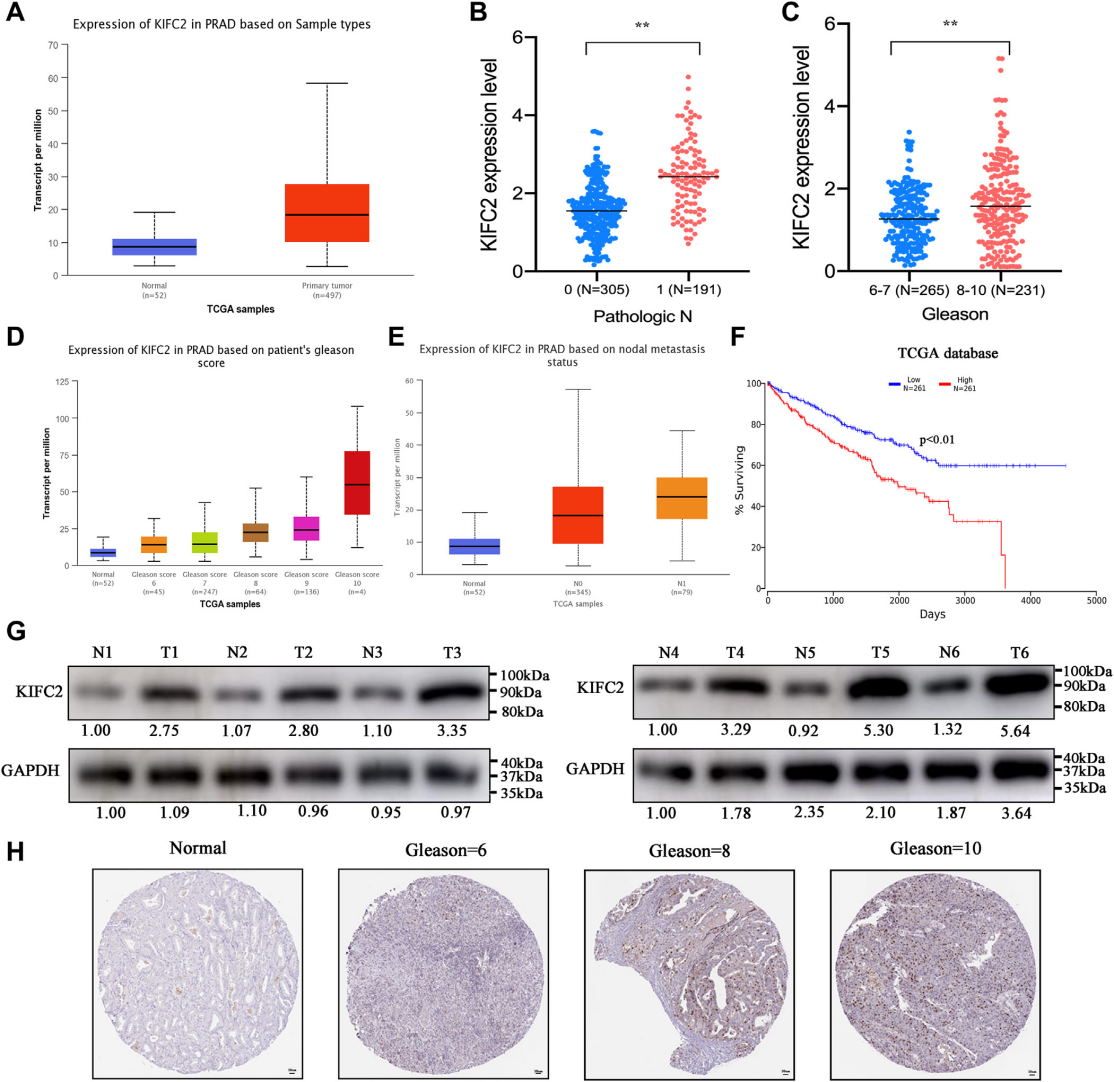
**KIFC2 was highly expressed in prostate cancer and associated with a poor prognosis.***A*, KIFC2 mRNA expression was investigated between PCa tissues and normal prostate tissues in TCGA database. *B*, comparison of KIFC2 expression levels between N classifications from TCGA database*. C* and *D*, TCGA database showed KIFC2 expression in PCa and correlated with Gleason scores. *E*, TCGA database showed KIFC2 expression in PCa and correlated with lymph nodes metastasis. *F*, survival curve was analyzed based on TCGA database. *G*, KIFC2 protein expression in PCa and normal tissues was detected *via* Western blot. *H*, representative IHC images for KIFC2 expression levels in PCa and normal prostate tissues. Data are mean ± SD (n = 3). ∗∗*p* < 0.01. IHC, immunohistochemistry; KIFC2, kinesin family member C2; PCa, prostate cancer; TCGA, The Cancer Genome Atlas.

We mined The Cancer Genome Atlas (TCGA) database and found that KIFC2 expression was elevated in PCa tissues when compared to normal tissues (Fig. 2*A*). In addition, the correlation of KIFC2 levels with clinical pathological characteristics was analyzed based on TCGA database, showing a positive correlation with the clinical N stages and Gleason scores (Fig. 2, *B* and *C* and Table 1), which was consistent with the analysis results based on UALCAN database (Fig. 2, *D* and *E*). Additionally, Western blot results showed that the protein levels of KIFC2 were significantly elevated in the PCa tissues (Fig. 2*G*). Analysis of PCa data from TCGA further suggested that KIFC2 was significantly correlated with the prognosis of PCa (Fig. 2*F*). Also, the immunohistochemical staining of tumor tissues also confirmed the elevated expression of KIFC2 in PCa with higher Gleason scores(Fig. 2*H*). These results reveal that KIFC2 is upregulated in PCa, and high KIFC2 expression is more likely to be in an advanced stage than those with low KIFC2 expression.

**Table 1:** Correlation between KIFC2 expression and clinicopathological characteristics of PCa

Characteristics total cases	N of case 496	KIFC2 expression		*p*
		Low (N = 230)	High (N = 266)	
Age (years)				
≤60	221	109 (21.98%)	132 (26.61%)	1.68E-01
>60	275	121 (24.4%)	134 (27.02%)	
Pathologic_M				
M0	442	224 (50.68%)	263 (58.31%)	6.39E-01
M1	9	(0.44%)	3 (0.22%)	
Pathologic_N				
N0	344	176 (41.61%)	168 (39.72%)	3.74E-02
N1	79	30 (13.30%)	49 (10.86%)	
Pathologic_T				
T2	187	90 (18.41%)	97 (19.84%)	6.61E-01
T3	291	124 (25.36%)	167 (34.15%)	
T4	11	3 (0.613%)	8 (1.64%)	
Gleason score				
6–7	60	14 (3.13%)	46 (10.29%)	3.84E-02
8–10	387	186 (41.61%)	201 (44.97%)	
Targeted molecular therapy				
Yes	53	37 (8.30%)	16 (3.59%)	6.74E-02
No	393	275 (61.66%)	118 (26.46%)	
Tumor recurrence				
Yes	59	43 (10.05%)	16 (3.74%)	8.49E-02
No	369	290 (67.76%)	79 (18.46%)	

### Targeting KIFC2 suppresses the proliferation and migration of PCa cells *in vitro*

**Figure 3:**
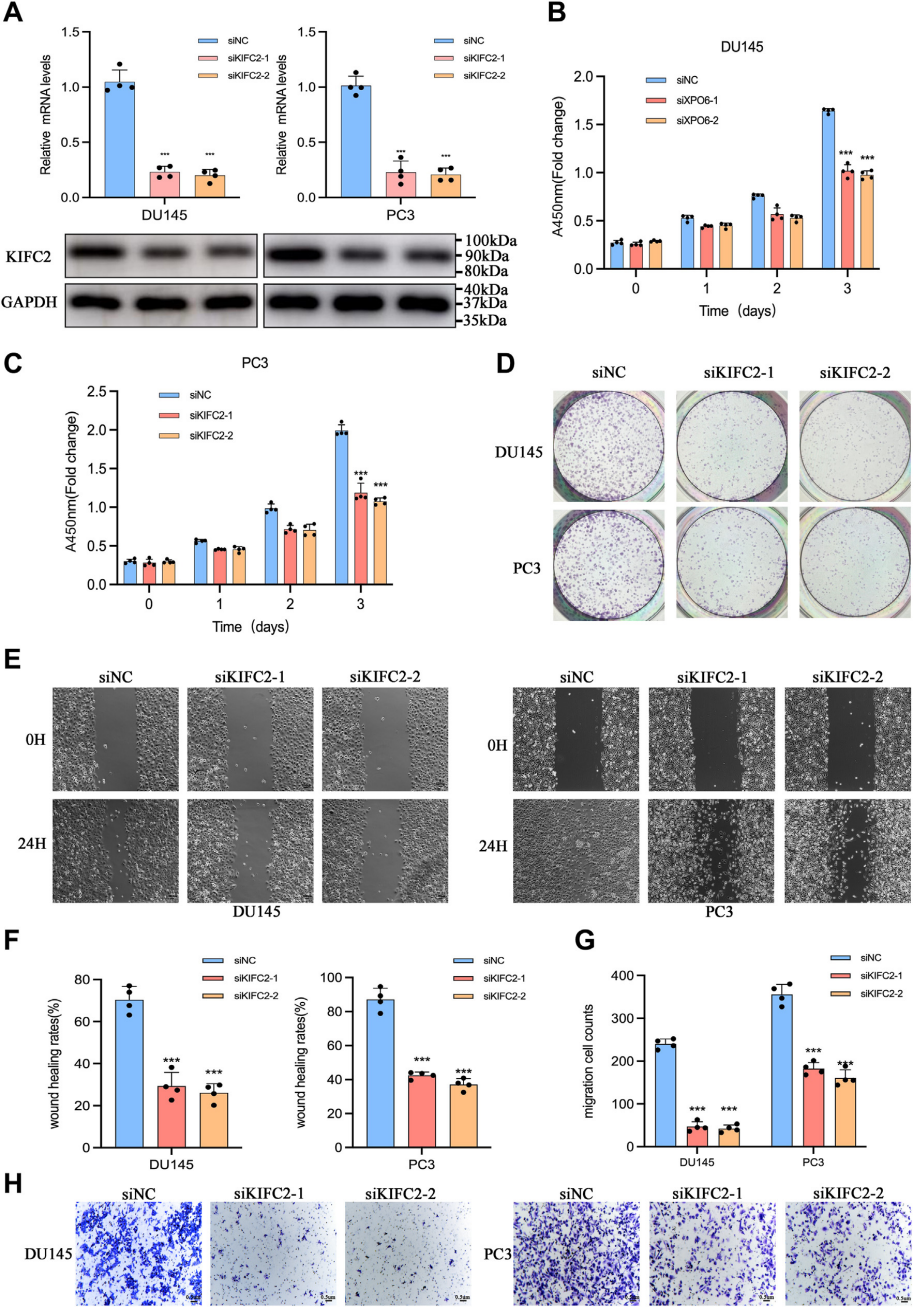
**KIFC2 promotes proliferation and migration of prostate cancer cells.***A*, Western blot and qRT-PCR analysis of KIFC2 knockdown efficiencies in PCa cells. *B*–*D*, the proliferation capabilities of PCa cells under KIFC2 knockdown were determined *via* CCK8 and colony-formation assays. *E* and *F*, representative images of wound-healing assays are presented. *G* and *H*, representative images of Transwell migration assays are presented. Data are mean ± SD (n = 3). ∗∗∗*p* < 0.001. CCK8, cell counting kit-8; KIFC2, kinesin family member C2; PCa, prostate cancer.

To investigate KIFC2 roles in biological behaviors of PCa cells, we silenced KIFC2 expression in DU145 and PC3 cells (Fig. 3*A*). Cell counting kit-8 (CCK8) assays showed that the viabilities of both cells significantly decreased (Fig. 3, *B* and *C*). Similar results were also observed in colony-formation assays (Fig. 3*D*). We next further evaluated the involvement of KIFC2 in the migration behaviors of the PCa cells using wound-healing assays and Transwell assays. Consistent with the effects of KIFC2 modulation on PCa cells proliferation, we found that knockdown of KIFC2 significantly decreased the migration abilities of both cells (Fig. 3, *E*–*H*).

### KIFC2 inhibition reduced Enza resistance in PCa cells

**Figure 4:**
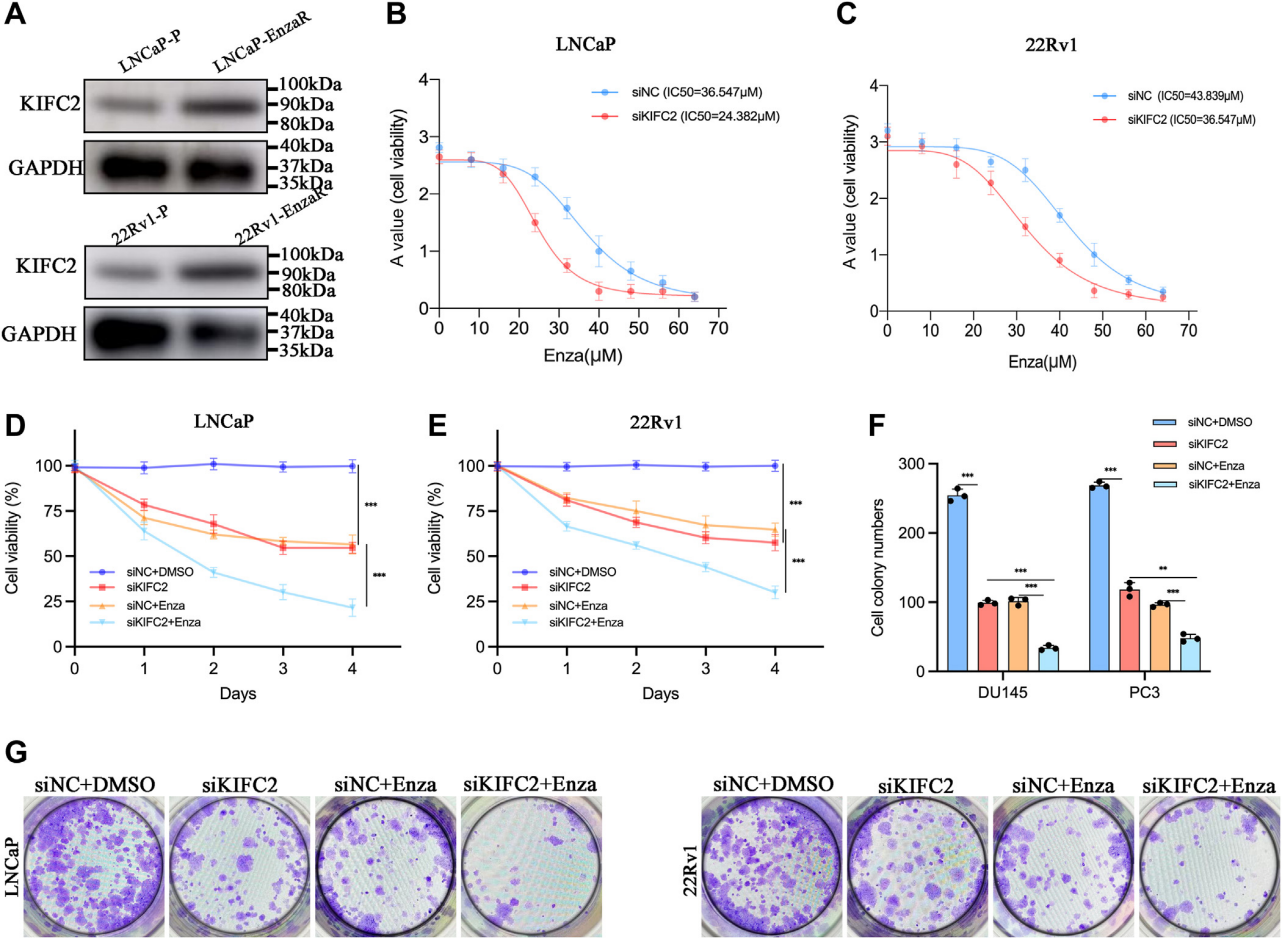
**KIFC2 influences the sensitivity of PCa cells to enzalutamide.***A*, the protein expression of KIFC2 in LNCaP-P/EnzaR and 22Rv1-P/EnzaR cells. *B* and *C*, CCK8 assays were used to measure the viability of the PCa cells treated with different concentrations of Enza for 24 h. *D* and *E*, CCK8 assays were used to assess cell viabilities of PCa cells after Enza intervention (15 μM) and/or siKIFC2 treatment. *F* and *G*, colony-formation assays were used to assess cell growth of PCa cells after Enza intervention and/or siKIFC2 treatment. Data are mean ± SD (n = 3). ∗∗*p* < 0.01, ∗∗∗*p* < 0.001. C2; Enza, enzalutamide; CCK8, cell counting kit-8; PCa, prostate cancer; KIFC2, kinesin family member.

The bioinformatic analysis results indicated that KIFC2 was associated with PCa progression and Enza resistance. Therefore, we decided to further verify the role of KIFC2 on Enza sensitivity to PCa. We constructed two Enza-resistance cells, LNCaP-EnzaR (LNCaP Enza resistance) and 22Rv1-EnzaR(22Rv1 Enza resistance), which all express AR, a key target of Enza. Indeed, the protein levels of KIFC2 was significantly increased in the LNCaP-EnzaR and 22Rv1-EnzaR cells than those in the parental LNCaP and 22Rv1 cells (Fig. 4*A*). CCK8 assays were performed to assess the cell viabilities of siKIFC2- and siNC-transfected LNCaP and 22Rv1 cells under various concentrations of Enza for 24 h. The IC50 values of the siKIFC2 group were significantly lower than that of the control group in both cells (Fig. 4, *B* and *C*). We explored whether Enza and KIFC2 knockdown functioned synergistically to suppress the proliferation of PCa cells. The combination of KIFC2 knockdown and Enza treatment revealed a stronger inhibitive effect on cell proliferation and growth in CCK8 assays (Fig. 4, *D* and *E*) and cell colony-formation assays (Fig. 4, *F* and *G*). Taking these findings together, it can be seen that the inhibition of KIFC2 could significantly enhance the therapeutic effect of Enza *in vitro*.

### Molecular signature regulated by KIFC2 in PCa

**Figure 5:**
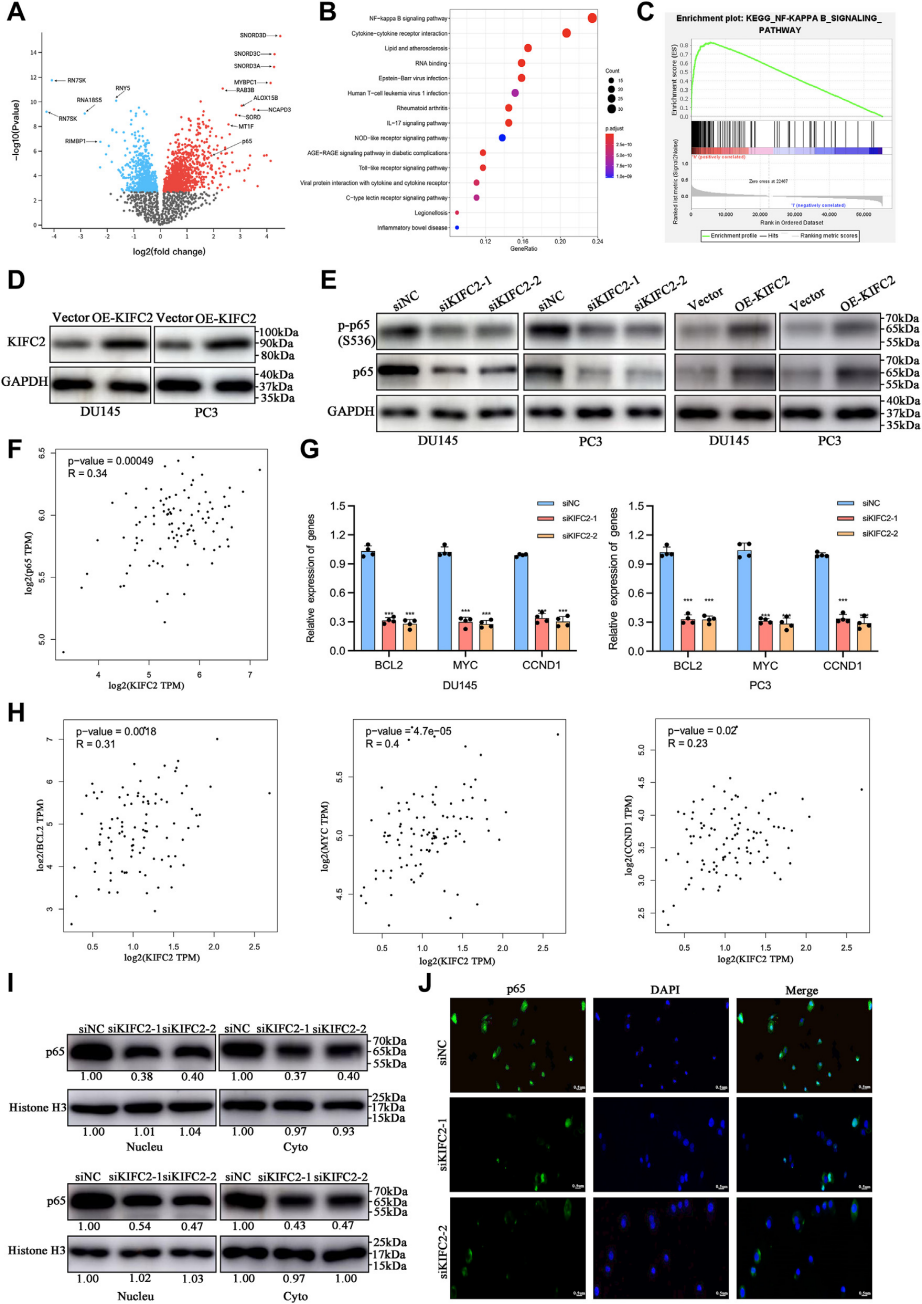
**KIFC2 regulates NF-κB pathway and promotes p65 protein expression.***A*, the differentially expressed genes from TCGA PRAD dataset was presented. *B*, the KEGG pathways for differentially expressed genes were presented. *C*, GSEA analysis of NF-κB pathway. *D*, the overexpression efficiencies of KIFC2 were assessed *via* Western blot. *E*, the total protein and phosphorylation levels of p65 were assessed *via* Western blot in KIFC2-knockdown or -overexpression PCa cells. *F*, correlation analysis between KIFC2 and p65 was conducted based on TCGA database. *G*, qRT-PCR was used to determine the target genes expression of NF-κB pathway. *H*, correlation analysis between KIFC2 and target genes of NF-κB pathway was conducted based on TCGA database. *I*, the nuclear or cytoplasmic protein levels of p65 were assessed *via* Western blot. *J*, IF analysis of the nuclear levels of the p65 in DU145 cell transfected with siNC or siKIFC2. Data are mean ± SD (n = 3). ∗∗∗*p* < 0.001. KIFC2, kinesin family member C2; TCGA, The Cancer Genome Atlas.

To determine the regulatory mechanism by which KIFC2 promotes cancer progression and chemoresistance in PCa, we performed functional analysis of differentially expressed genes associated with KIFC2 based on TCGA database. After comparison, 331 of the differentially expressed genes were upregulated, and 216 were downregulated (Fig. 5*A*). Then, KEGG enrichment analysis revealed several pathways significantly enriched. Among them, the NF-κB pathway ranked first (Fig. 5*B*). The GSEA analysis also showed that KIFC2 expression was positively correlated with the NF-κB pathway (Fig. 5*C*). To validate above hypothesis, we examined protein expression and phosphorylation levels of NF-κB p65 in PCa cells with KIFC2 knockdown or overexpression. The efficiencies of KIFC2 overexpression were assessed *via* Western blot (Fig. 5*D*). We found knockdown of KIFC2 inhibited the protein expression and phosphorylation levels of NF-κB p65, while KIFC2 overexpression enhanced that of NF-κB p65 (Fig. 5*E*), which were consistent with the positive correlation results *via* correlation analysis based on TCGA database (Fig. 5*F*). We also examined the expression of target genes of NF-kB pathway, including BCL2, MYC, and CCND1, which are also associated with tumor proliferation. KIFC2 knockdown inhibited the expression levels of these genes significantly (Fig. 5*G*), which were consistent with the positive correlation results *via* correlation analysis based on TCGA database (Fig. 5*H*), confirming that KIFC2 regulates the NF-kB pathway.

We also further examined the nuclear expression of NF-κB p65 in KIFC2-knockdown PCa cells, as NF-κB p65 is a known nuclear transcription factor which enters the nucleus upon activation. As shown in Figure 5*I*, knockdown of KIFC2 reduced NF-κB p65 protein levels in the nucleus. This result was supported by immunofluorescence analysis, which showed a decreased nuclear accumulation of NF-κB p65 in KIFC2-knockdown PCa cells (Fig. 5*J*). Together, these results suggest that decreased levels of KIFC2 inactivated the NF-κB pathway.

### KIFC2 promotes PCa progression and Enza resistance *via* p65

**Figure 6:**
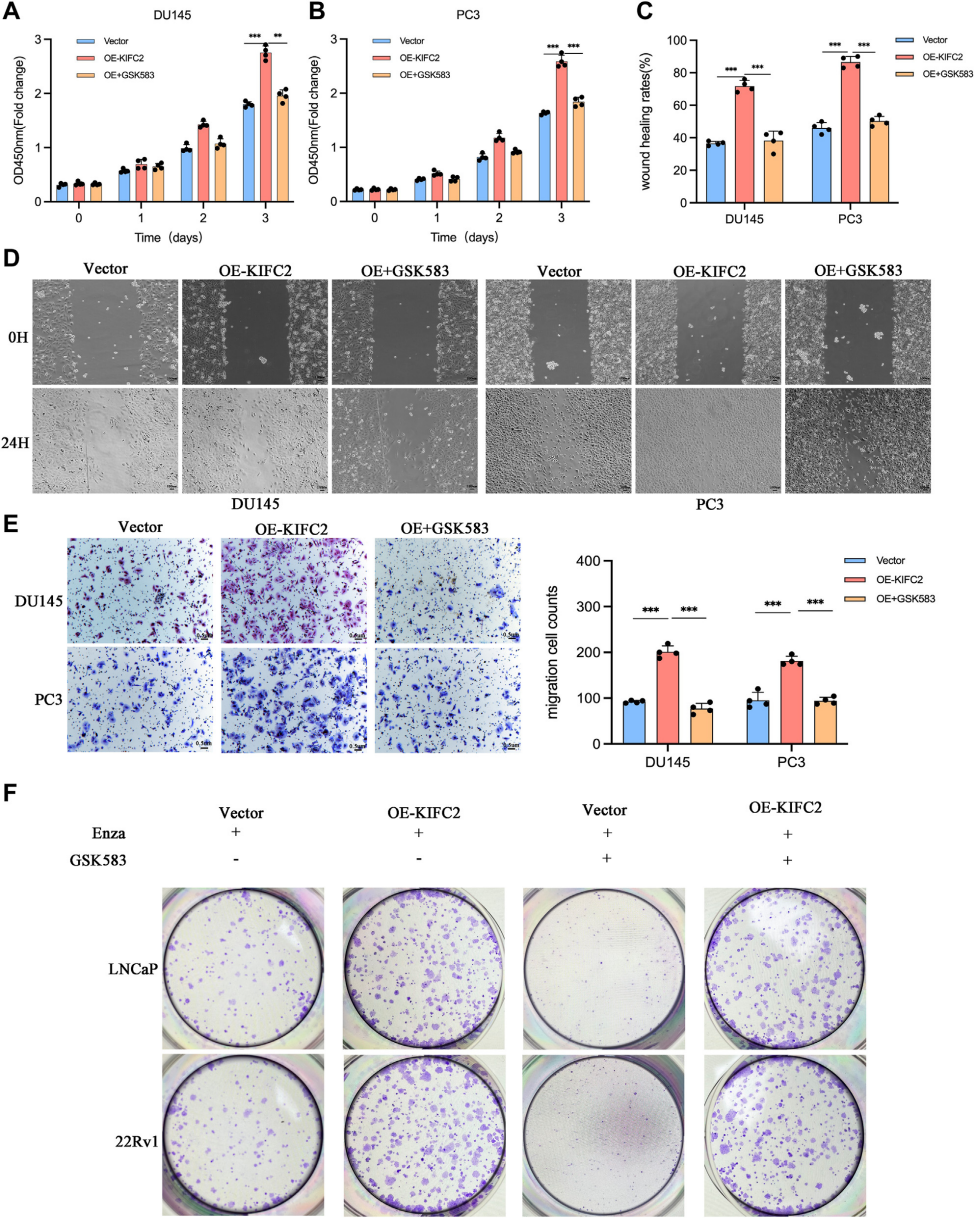
**KIFC2 modulates PCa progression and enzalutamide resistance *via* NF-κB pathway.***A* and *B*, CCK8 assays showed that NF-κB pathway inhibitor GSK583 reversed KIFC2-induced promotion of proliferation in PCa cells. *C*–*E*, wound-healing and Transwell migration assays showed that GSK583 reversed KIFC2-induced promotion of migration in PCa cells. *F*, colony formation assays showed that GSK583 reversed KIFC2-induced enhancement of resistance to enzalutamide. Data are mean ± SD (n = 3). ∗∗*p* < 0.01, ∗∗∗*p* < 0.001. CCK8, cell counting kit-8; KIFC2, kinesin family member C2; PCa, prostate cancer.

The NF-κB pathway has been shown to implicate in the tumor initiation and maintenance and mediate chemoresistance in PCa (19). In view of the regulatory role of KIFC2 on the NF-κB pathway, we wondered whether NF-κB pathway can mediate the protumorigenesis and proresistance activities of KIFC2. To confirm that KIFC2 induces tumor progression and Enza resistance dependent on NF-κB p65, we detected the effect of Enza on the proliferation of KIFC2-overexpressing PCa cells pretreated with NF-κB chemical inhibitors GSK583. We found that GSK583 could enhance the cytotoxic effect of Enza on KIFC2-overexpression PCa cells (Fig. 6*F*). In addition, we also found GSK583 could rescue the malignant phenotypes enhanced by KIFC2 overexpression assessed by CCK8, wound-healing, and Transwell migration assays (Fig. 6, *A*–*E*).

### Knockdown of KIFC2 inhibits PCa cell growth *in vivo*

**Figure 7:**
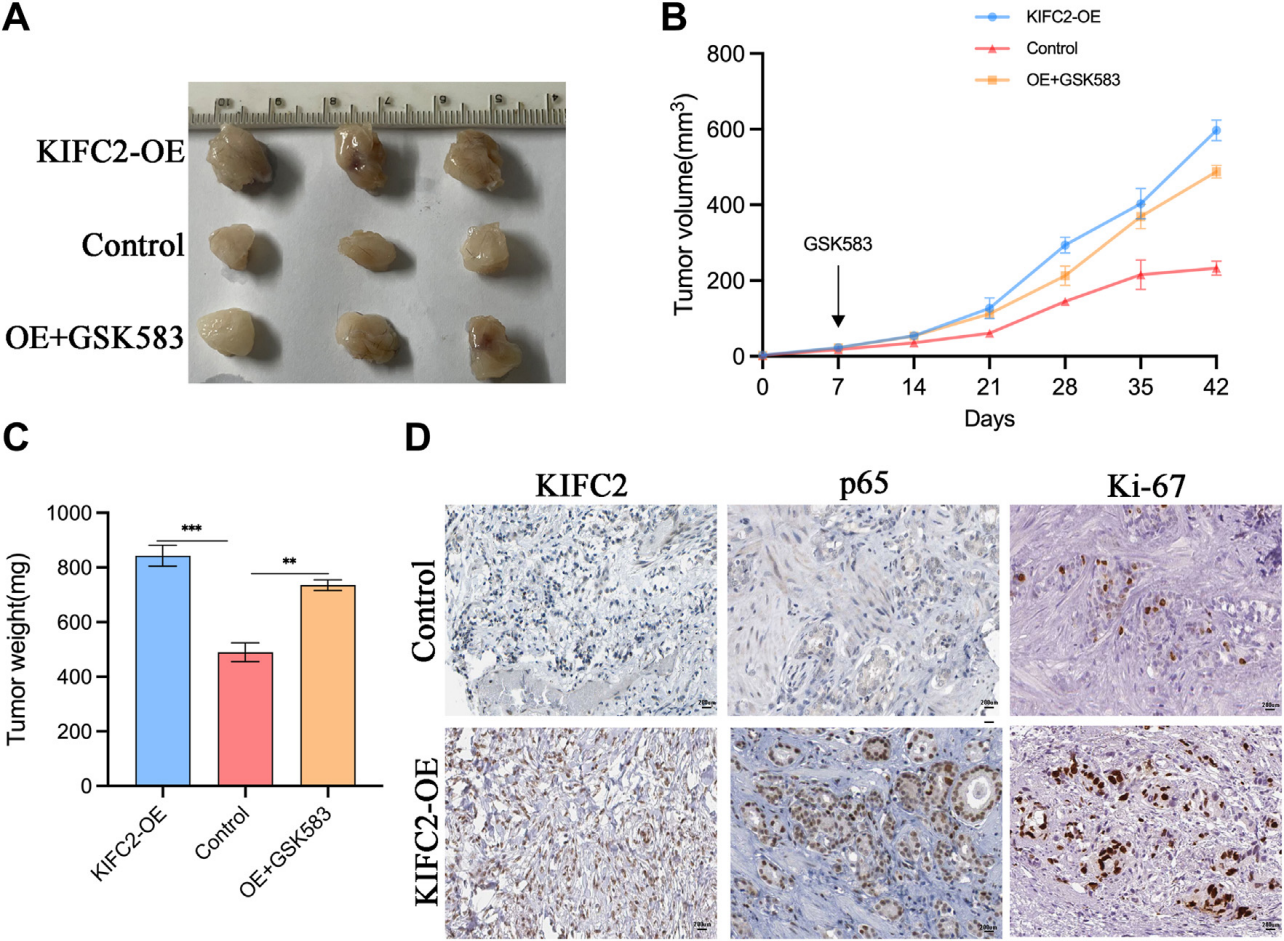
**KIFC2 depletion inhibits PCa cell growth *in vivo*.***A*, PC3 cells transfected with shKIFC2 with or without GSK583 treatment. *B*, the sizes of tumor xenografts were measured every 7 days. *C*, the tumor masses were harvested from nude mice at the experimental endpoint. *D*, KIFC2, p65, and Ki-67 expression levels in tumor tissues were measured *via* IHC analysis. Data are mean ± SD (n = 3). ∗∗*p* < 0.01, ∗∗∗*p* < 0.001. IHC, immunohistochemistry; KIFC2, kinesin family member C2; PCa, prostate cancer.

To investigate the effects of KIFC2 on PCa growth *in vivo*, a xenograft mouse model was established by subcutaneously injecting PC3 cell that had been transfected with lentivirus harboring vector or KIFC2 into BALB/c nude mice and treated with or without GSK583. *In vivo* studies showed that KIFC2-overexpression mice experienced significant increase in tumor volumes and weights than control groups, while GSK583 treatment partially reversed this promoting effect (Fig. 7, *A*–*C*). Subsequently, the expressions of KIFC2, NF-κB p65, and Ki-67 in control and KIFC2-OE groups of transplanted tumors were examined by performing immunohistochemical staining. The immunohistochemistry showed that the KIFC2, NF-κB p65, and Ki-67 expression levels of KIFC2-transduced PC3 xenografts significantly increased than vector-transfected xenografts (Fig. 7*D*). Taken together, these findings indicate that KIFC2 could promote PCa growth *in vivo*.

## Discussion

PCa progression and drug resistance are major limitations to PCa patients’ survival. Genomic aberrations that confer resistance to Enza have been identified in numerous studies, including alterations of the AR, DNA repair, cell cycle, PI3K-AKT-mTOR, and Wnt-β-catenin pathways (20). Therefore, the modulation of genes that regulate above pathways to sensitize the effects of Enza against CRPC has been the focus of recent research (21, 22, 23, 24).

Kinesin-14 motor proteins participate in massed physiological processes, including cell cycle regulation, apoptosis, DNA repair, tumorigenesis, and chemotherapeutic resistance (25, 26, 27, 28). However, the clinical function, mechanism, and/or targets of KIFC2 in PCa remain unknown. Here, we demonstrated that KIFC2 was highly expressed in PCa tissues and associated with Gleason scores. Subsequently, a series of experiments were designed to demonstrate the regulation of KIFC2 on biological functions of PCa cells. Next, we found that KIFC2 was associated with the NF-κB pathway and was especially correlated with NF-κB p65 from the KEGG and GSEA database analysis. Furthermore, Western blotting analysis showed that targeting KIFC2 could downregulate NF-κB p65 protein expression and nuclear translocation. Additionally, to confirm whether KIFC2 promoted the PCa progression and Enza resistance by NF-κB p65, we pretreated KIFC2-overexpressing PCa cells with NF-κB chemical inhibitors GSK583. As expected, NF-κB pathway inhibition markedly reversed the enhanced malignant phenotype induced by KIFC2 overexpression *in vitro* and *in vivo*. These results confirmed that KIFC2 regulated PC3 progression by NF-κB pathway and provided a potential target for PCa treatment.

However, this study has some limitations. The upstream regulator of KIFC2 is still unclear. Also, we do not know whether this occurs in other human malignancies. In addition, how does KIFC2 regulate NF-κB p65 protein level and nuclear translocation needs to be clarified. Further studies are required to address these limitations.

In conclusion, clinical PCa samples, cell-based experiments, and nude mouse models were employed to provide the first evidence that KIFC2 mediate PCa progression and chemotherapeutic resistance by activating NF-κB pathway. KIFC2 may be a novel molecular target for PCa treatment.

## Experimental procedures

### Data mining

We analyzed three GEO datasets (GSE69249, GSE70770, and GSE70768) to identify drug resistance and CRPC-related genes and further analyzed differential expression of KIFC2 (https://www.ncbi.nlm.nih.gov/geo). Clinical and prognosis data of PCa patients were obtained from TCGA database.

### Cell cultures and treatment

PCa cell lines (LNCaP, 22Rv1, DU145, and PC3) were purchased from American Type Culture Collection (ATCC). Cells were cultured in the RPMI-1640 supplemented with 1% penicillin/streptomycin and 10% FBS in a 37 °C water-saturated 5% CO_2_ atmosphere. Enza-resistant LNCaP cell (LNCaP-EnzaR) and Enza-resistant 22Rv1 cell (22Rv1-EnzaR) were kindly provided by Yu Lin (Shanghai Jiao Tong University School of Medicine). Enza (#HY-70002) and GSK583 (#HY-100339) was obtained from MedChemExpress and handled according to the manufacturer's recommendations. The cells were treated with Enza for 24 h. After Enza treatment, follow-up assays were performed.

### Cells transfection and lentiviral constructs

Cells were transfected using Lipofectamine 2000 reagent (Invitrogen) according to the manufacturer's instructions. Two siRNAs targeting KIFC2 were designed and purchased from GenePharma Technology. The sequences of siRNAs used in this study are as follows: siNC: TTCTCCGAACGTGTCACGT; siKIFC2-1; AGCAUGGUGGAGAUCUACATT; and siKIFC2-2: CGUUGCUCAUCUACAUCUUTT. Full-length KIFC2 and KIFC2-shRNA were cloned into PCDH-CMV-EF1A-T2A-PURO and PGMLV-hU6-MCS-CMV-ZsGreen1-PGK-Puro-WPRE vectors, respectively. Transient transfection of KIFC2-expressing vector into cells was achieved using the Lipofectamine 2000 reagent according to the protocol. To generate stable knockdown cell lines, lentiviral particles equipped with shRNAs targeting KIFC2 were purchased from GenePharma Technology. KIFC2-shRNA oligonucleotide sequences are as follows: shNC: TTCTCCGAACGTGTCACGT; shKIFC2: AGGCUACAGCGUCUGCAUCTT. Stable cell lines were obtained after selection with 2.5 μg/ml puromycin (Beyotime) for 10 days. The efficiencies of KIFC2 knockdown and overexpression were evaluated by Western blot and qRT-PCR.

### RNA isolation and qRT-PCR

Total RNA was isolated using RNAiso plus (Takara) and reverse transcription was performed using PrimeScript RT reagent kit with gDNA Eraser (Japan) according to the manufacturer’s protocol. The cDNAs were quantified by the qRT-PCR using TB Green *Premix Ex Taq* II (Takara). Glyceraldehyde-3-phosphate dehydrogenase or U6 was used as an endogenous control for mRNA or miRNA detection, and the fold change was calculated with the relative quantification method (2^−ΔΔCt^). Primers used to detect mRNA expression are shown in Table S1.

### Western blot

Briefly, breast cancer cells, after transfection or without processing, were harvested and lysed by using a RIPA lysis buffer (Beyotime). Protein concentration was determined with the BCA protein assay kit (Beyotime). Then, the protein was separated on SDS-PAGE gels and transferred to PVDF membranes (Millipore). Membranes were blocked by 5% nonfat milk, then incubated with primary antibodies overnight at 4 °C and with corresponding secondary antibodies afterward. Detailed information about the antibodies used in this study was shown as follows: KIFC2 (PA5-40813; Invitrogen), NF-κB p65 (ab16502; Abcam), p-p65 (phospho S536) (ab76302), glyceraldehyde-3-phosphate dehydrogenase (ab8226; Abcam), Histone H3 (BS1174; Bioworld). Protein bands were visualized with ECL.

### Proliferation assays

For CCK8 assay, the cell suspension was prepared with complete medium and inoculated into 96-well plates (3000 cells/well). After culture for 24 h, the CCK8 solution (Dojindo) was added to each well and incubated at 37 °C for 60 min. Finally, absorbance was measured at 450 nm using a microplate reader. For colony formation assay, after 2 weeks of culturing the PCa cells, the plates were washed twice with PBS. Next, we fixed the cells with paraformaldehyde and wash the plates twice with PBS. Finally, the cells were stained with crystal violet for 10 min, and the plates were washed twice with PBS and dried thereafter.

### Cell migration assays

For wound-healing assay, the transfected PCa cells were cultured in 12-well plates to 90% density. Then, the cells were vertically scratched using a 200 ml pipette, and the scratched cells were then washed with PBS. 2 ml of culture medium was then added for further cultivating the cells for 24 h. Finally, the cells were observed to calculate the average wound gap between wound edges. For Transwell migration assay, 5 × 10^4^ cells in 200 μl serum-free medium were seeded into Transwell inserts (Corning) and placed in 24-well plates with 600 μl medium containing 20% FBS. After 24 h incubation, the cells were fixed with 4% PFA and stained with crystal violet. Finally, the samples were observed for recording images of cell migration.

### Immunofluorescence

The cell immunofluorescence staining was performed following the manufacturer's instructions. Primary antibodies against NF-κB p65 (ab32536; Abcam) were applied at a ratio of 1:200. Fluorescent secondary antibodies (Proteintech) were also applied at a ratio of 1:200. Image capture was performed by an inverted fluorescence microscope.

### Immunohistochemistry staining

The paraffin blocks were sliced into 4-μm-thick sections, and the process of section dewaxing was performed in xylene and different concentrations of ethanol, incubated with primary antibodies at 4 °C overnight. The primary antibodies used are as follows: KIFC2 (PA5-40813; Invitrogen), NF-κB p65 (ab16502; Abcam), Ki-67 (ab15580; Abcam). Then the sections were incubated with generally biotinylated goat anti-rabbit serum and streptavidin–peroxidase conjugate for 15 min at room temperature and finally stained with diaminobenzidine.

### Xenograft assay

Nude mice (4-weeks-old) were purchased from Shanghai Jihui Experimental Animal Feeding Co Ltd. Subcutaneous tumor growth assays were performed with Control-, KIFC2-OE-, and OE+GSK583-treated PC3 cell. At the end point, all mice were sacrificed, and the tumors were excised and weighed. All experimental procedures followed the strict NIH guidelines and acquired ethical approval from Loudi City Central Hospital for Animal Research.

### Statistical analysis

All statistical analyses were performed in GraphPad Prism 8.0 software. The number of technical replicates, biological replicates, and independent experiments performed are indicated in the figure legends. Statistical analyses were by a two-tailed Student’s *t* test. Data are presented as mean ± standard error of the mean for analyses, and results were considered statistically significant with *p* < 0.05, ∗*p* < 0.05, ∗∗*p* < 0.01, ∗∗∗*p* < 0.001.

## Data availability

The datasets used and/or analyzed during the current study are available from the corresponding author on reasonable request.

## Supporting information

This article contains supporting information.

## Conflict of interest

The authors declare that they have no conflicts of interests with the contents of this article.
