# Supplementary material for: The kinesin-14 family motor protein KIFC2 promotes prostate cancer progression by regulating p65
Source: J Biol Chem. 2023 Sep 14;299(11):105253. doi: 10.1016/j.jbc.2023.105253 (PMC10590982; doi:10.1016/j.jbc.2023.105253)
Supplement: Supporting Table S1 [file mmc1.docx]

| **Table S1.** Sequences of primers used for qRT-PCR in this study | | |
| --- | --- | --- |
| Gene | Sequence | |
| KIFC2 | Forward | 5′- AAGGGAAATATCCGTGTGCTG -3′ |
|  | Reverse | 5′- GTCTAGGCGGAATCGACGATG -3′ |
| BCL2 | Forward | 5′- GGTGGGGTCATGTGTGTGG -3′ |
|  | Reverse | 5′- CGGTTCAGGTACTCAGTCATCC -3′ |
| MYC | Forward | 5′- GGCTCCTGGCAAAAGGTCA -3′ |
|  | Reverse | 5′- CTGCGTAGTTGTGCTGATGT -3′ |
| CCND1 | Forward | 5′- GCTGCGAAGTGGAAACCATC -3′ |
|  | Reverse | 5′- CCTCCTTCTGCACACATTTGAA -3′ |
| GAPDH | Forward | 5′- CTGGGCTACACTGAGCACC -3′ |
|  | Reverse | 5′- AAGTGGTCGTTGAGGGCAATG -3′ |
