# Supplementary material for: The kinesin-14 family motor protein KIFC2 promotes prostate cancer progression by regulating p65
Source: J Biol Chem. 2023 Sep 14;299(11):105253. doi: 10.1016/j.jbc.2023.105253 (PMC10590982; doi:10.1016/j.jbc.2023.105253)

Figure 3A


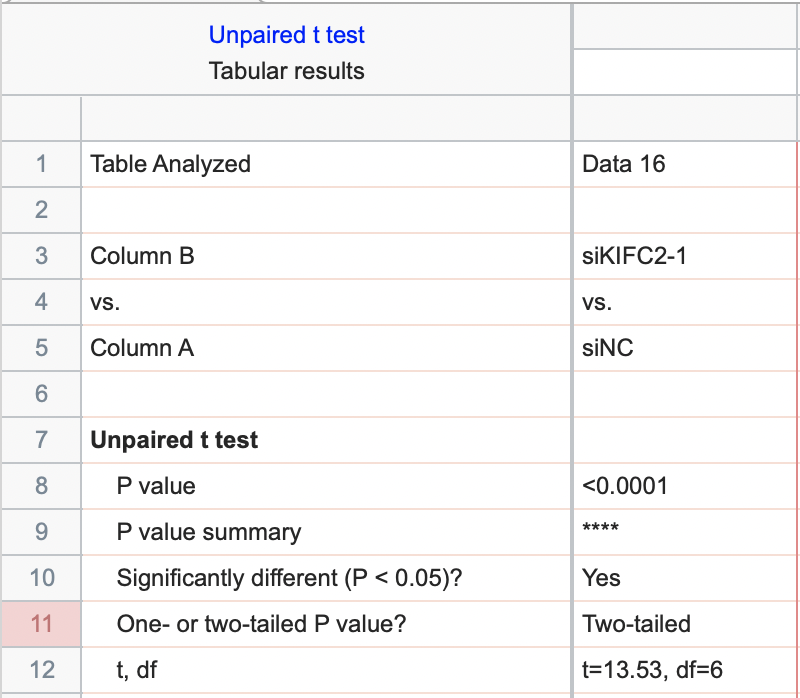

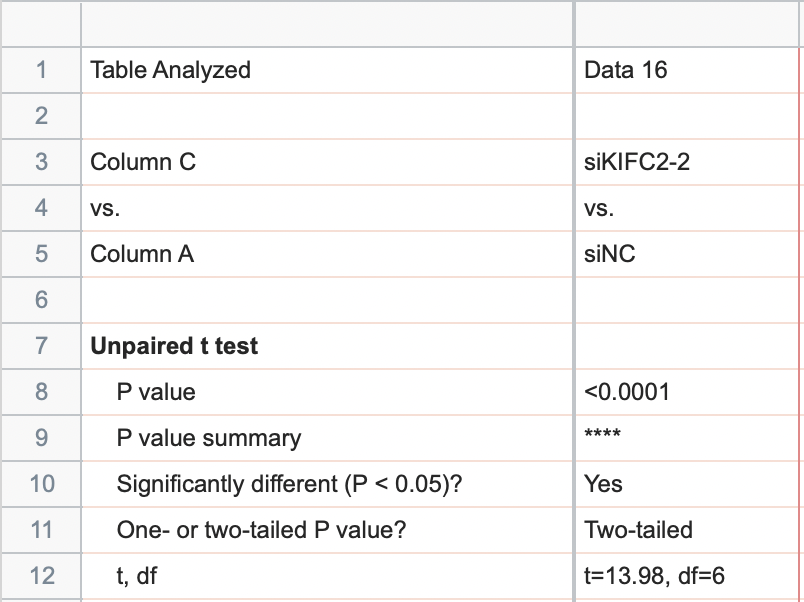


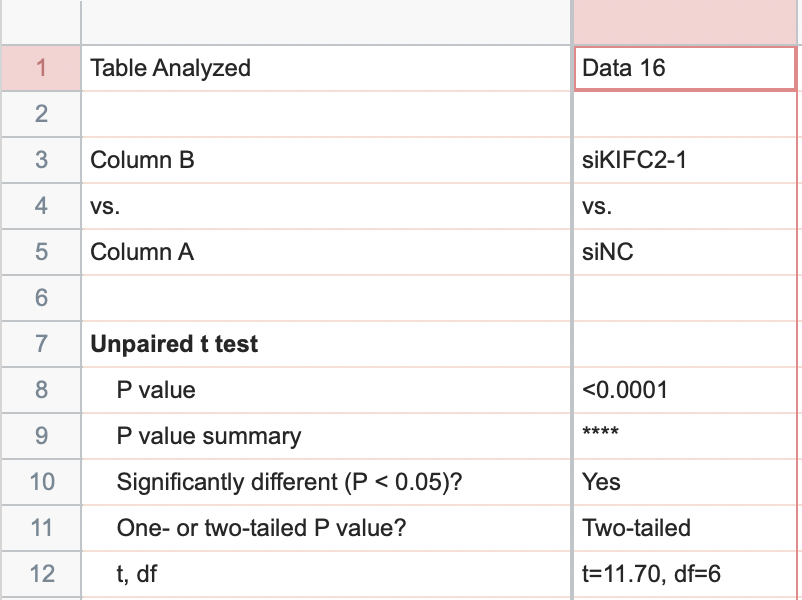

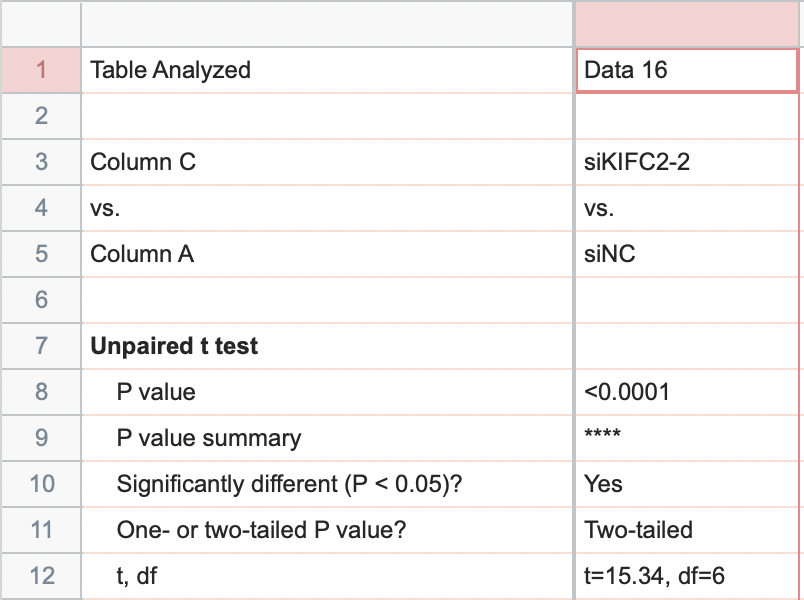


Figure 3B


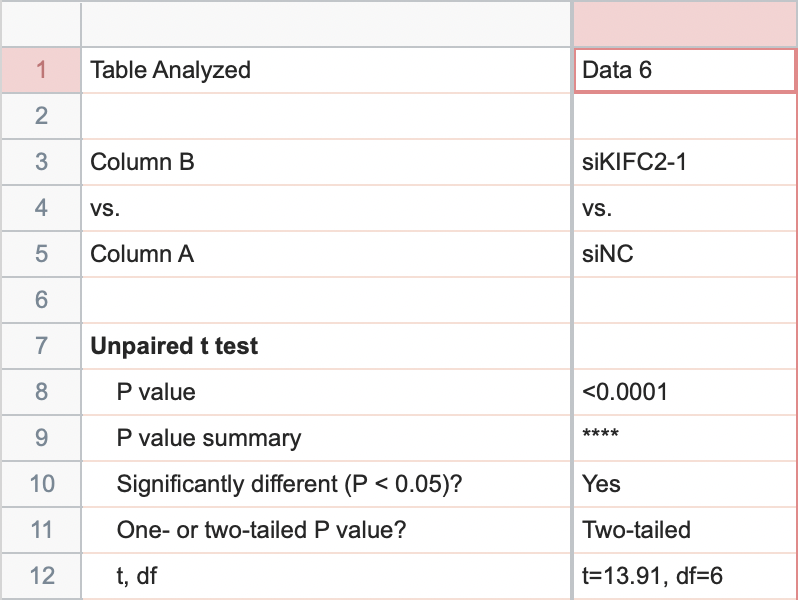

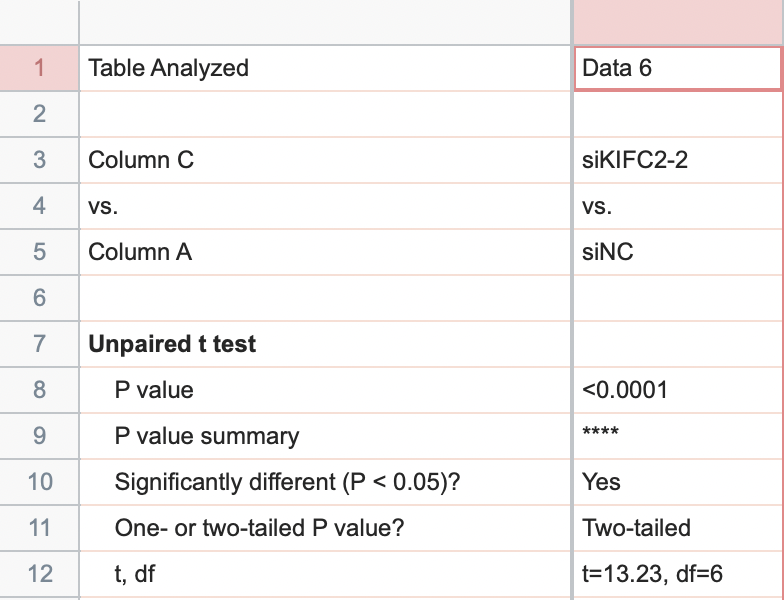


Figure 3C


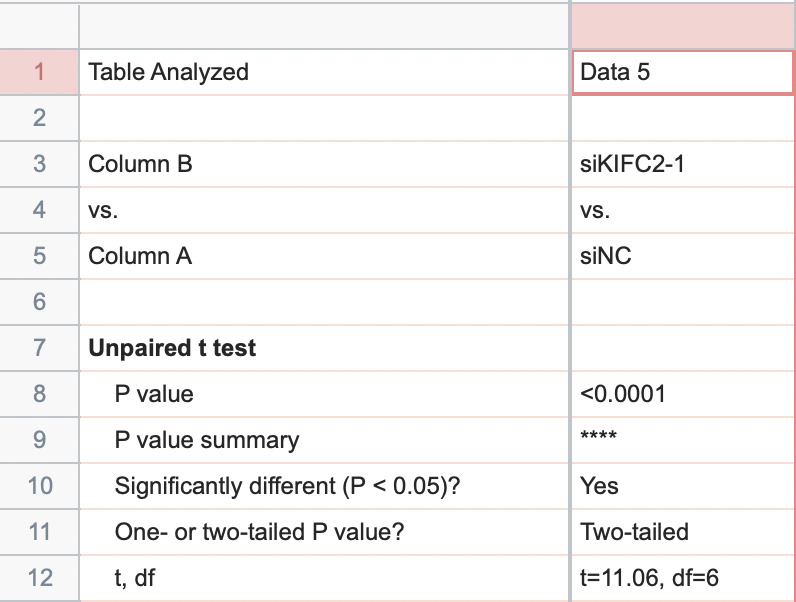

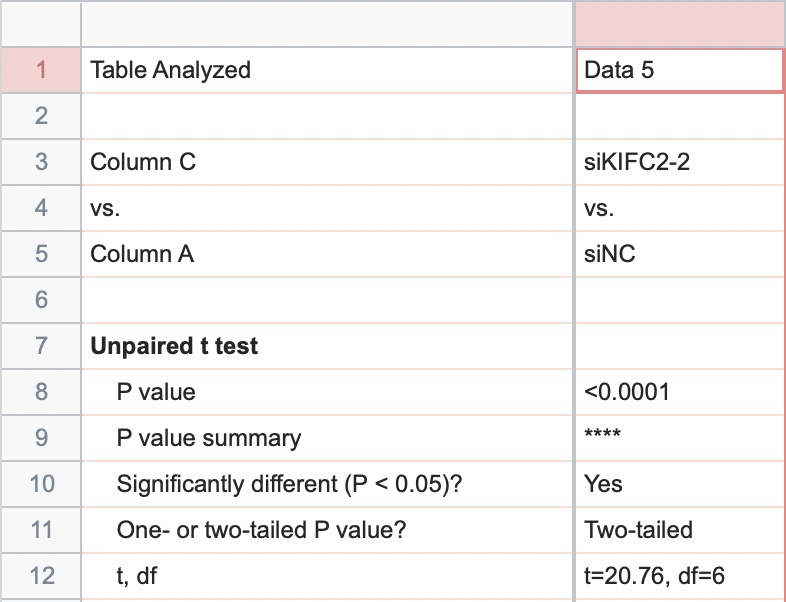


Figure 3F


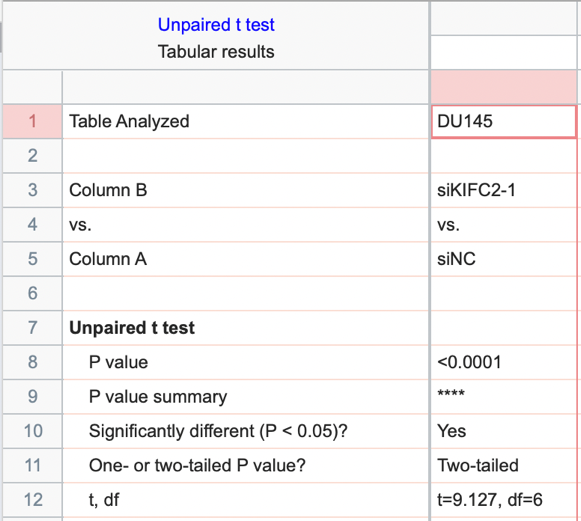

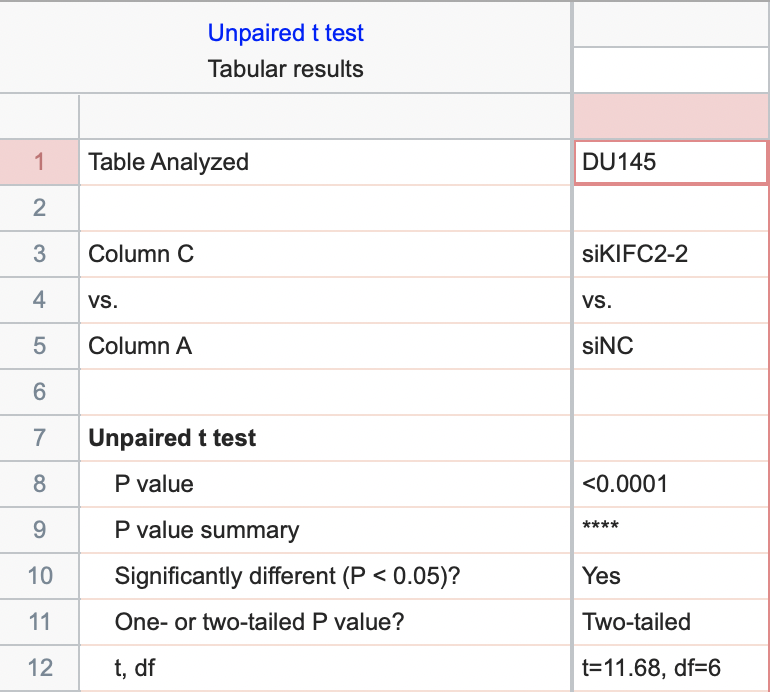


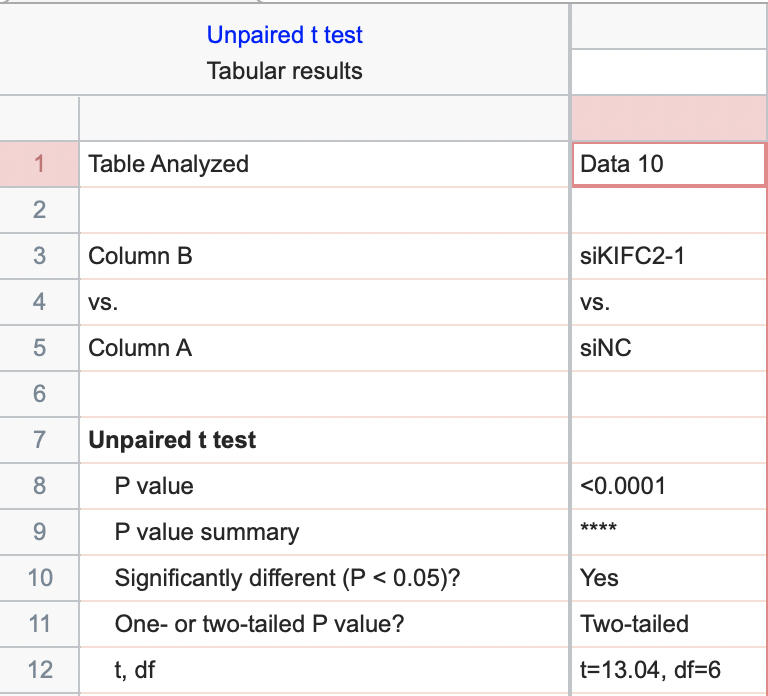

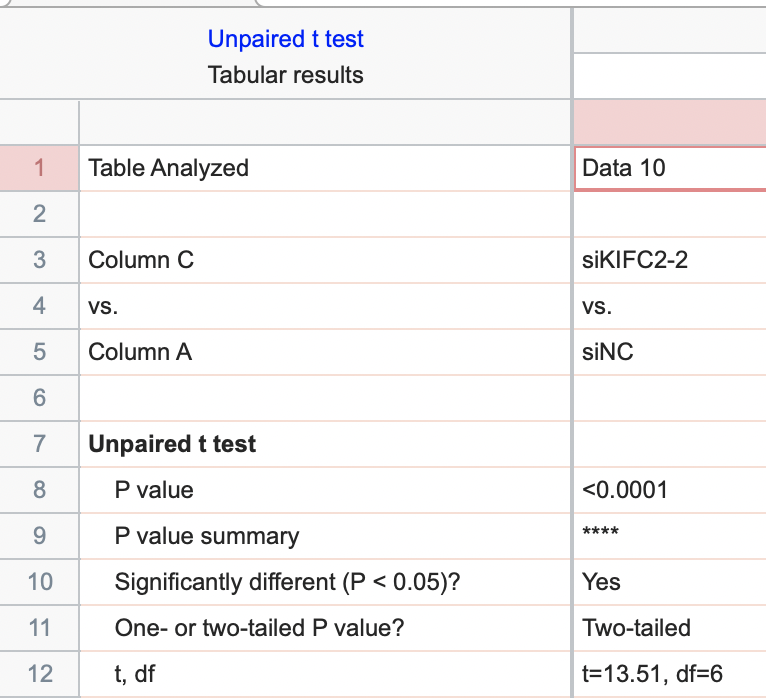


Figure 3G


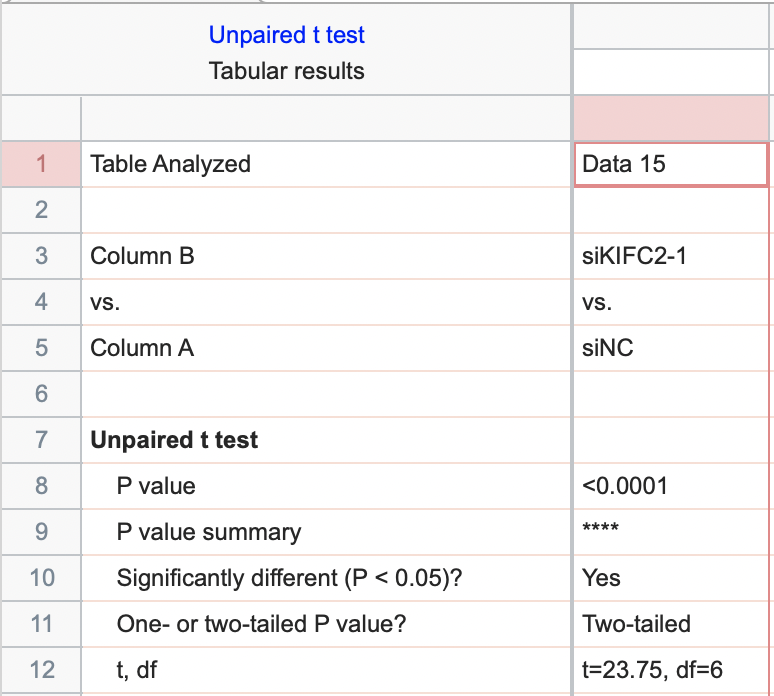

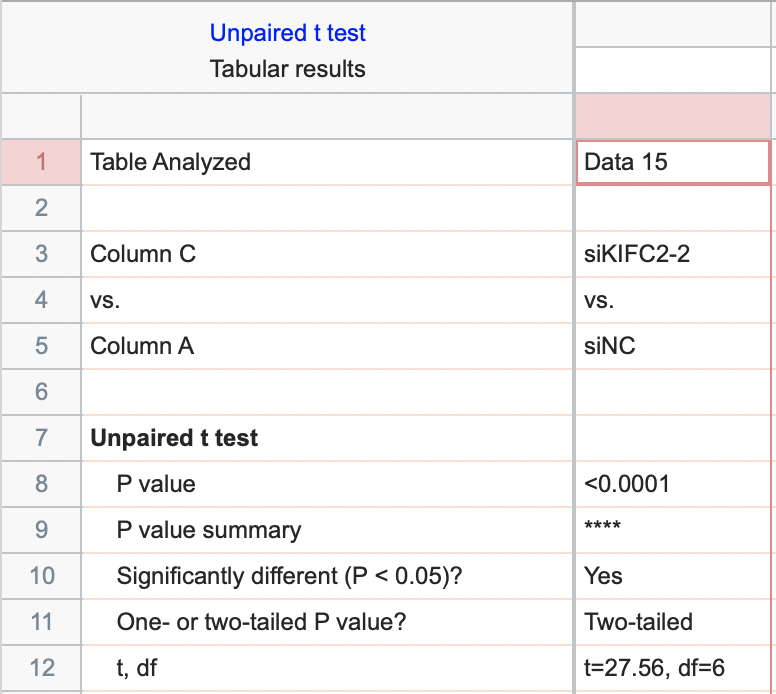


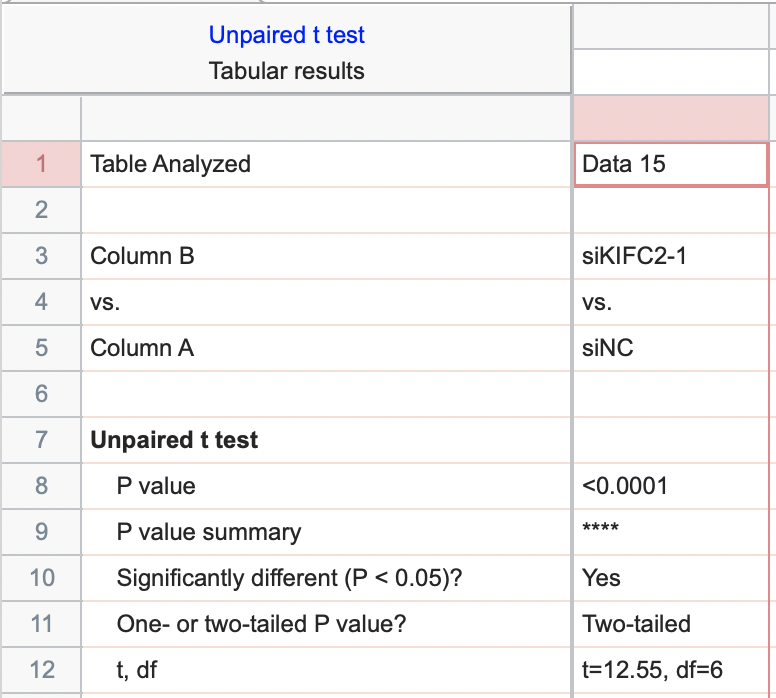

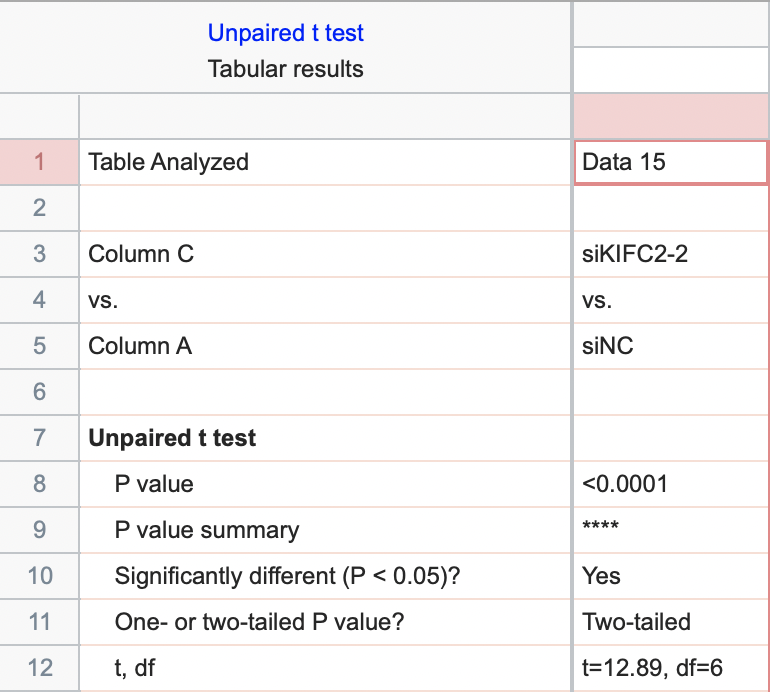


Figure 4F


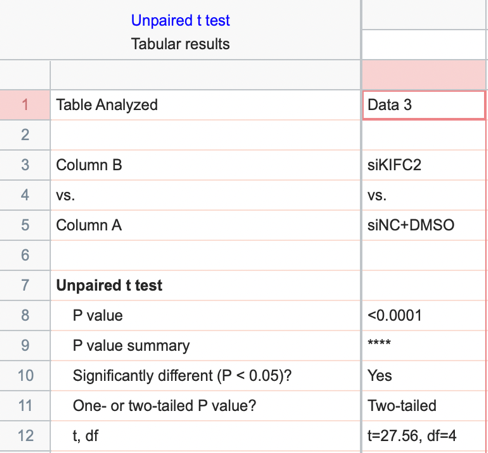

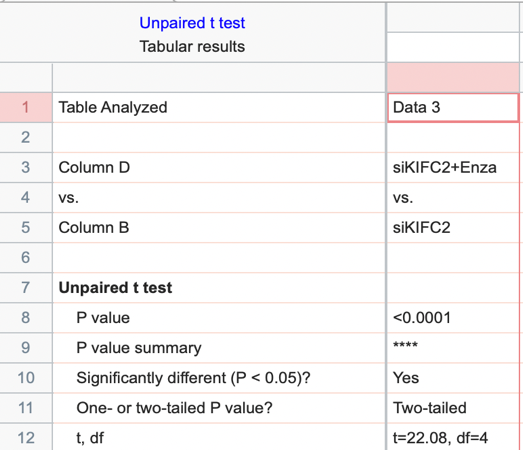

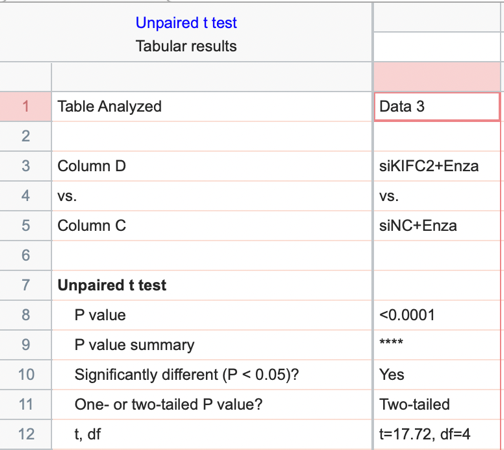


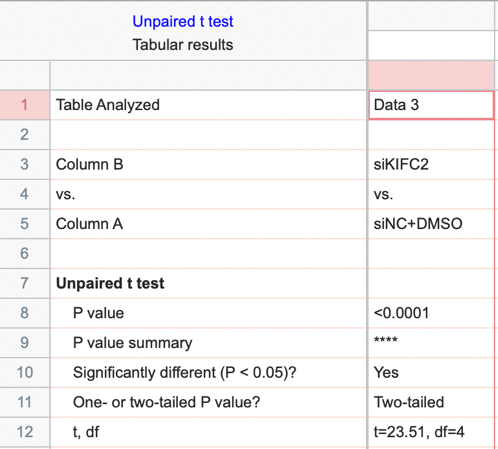

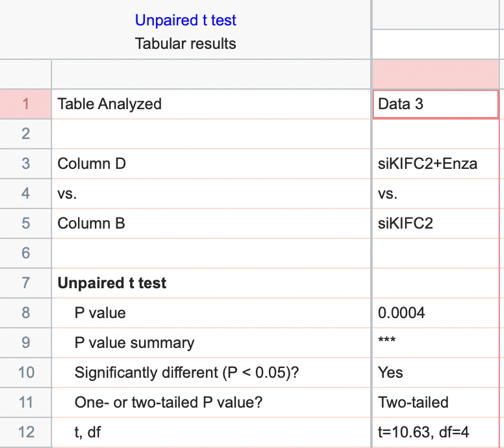

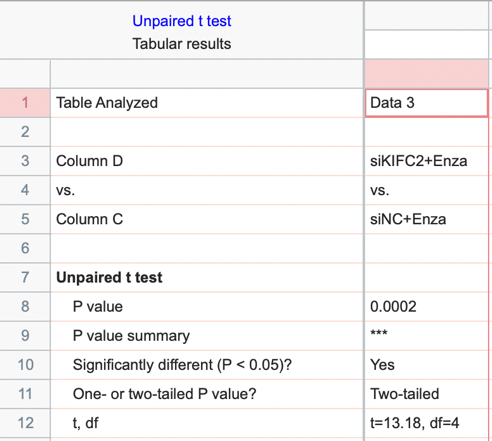


Figure 5G-DU145


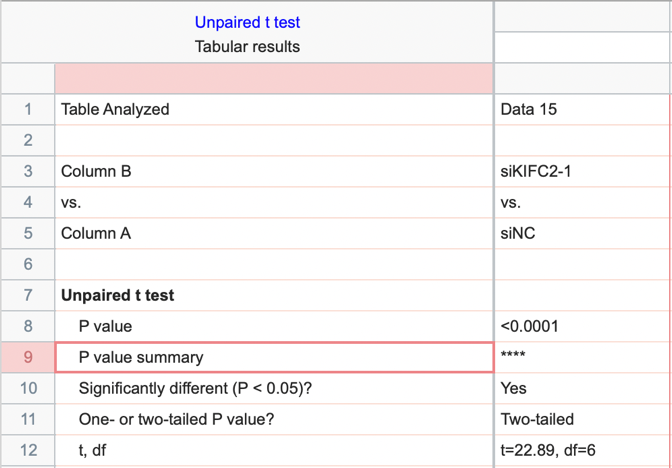

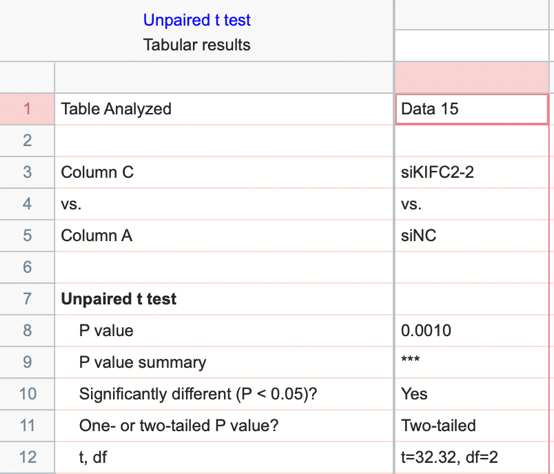


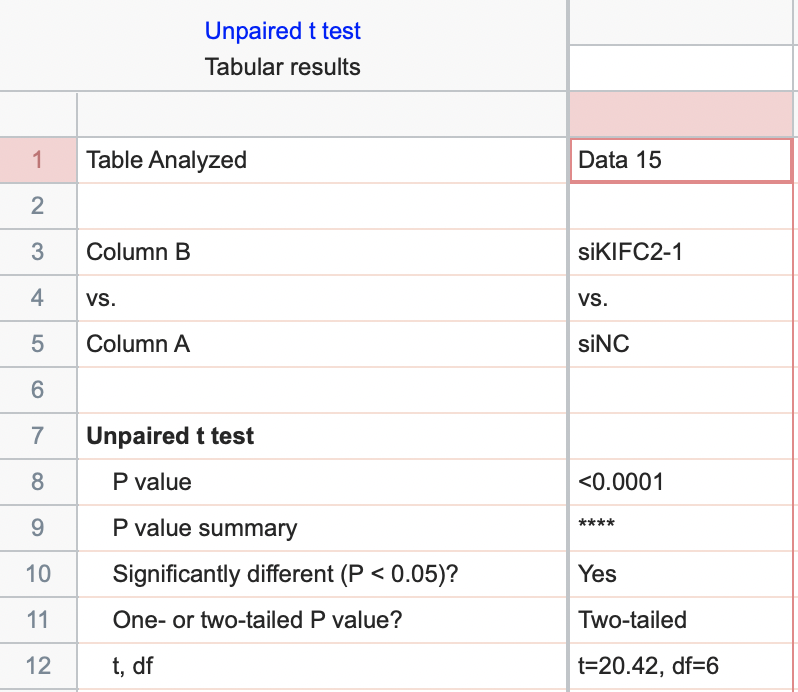

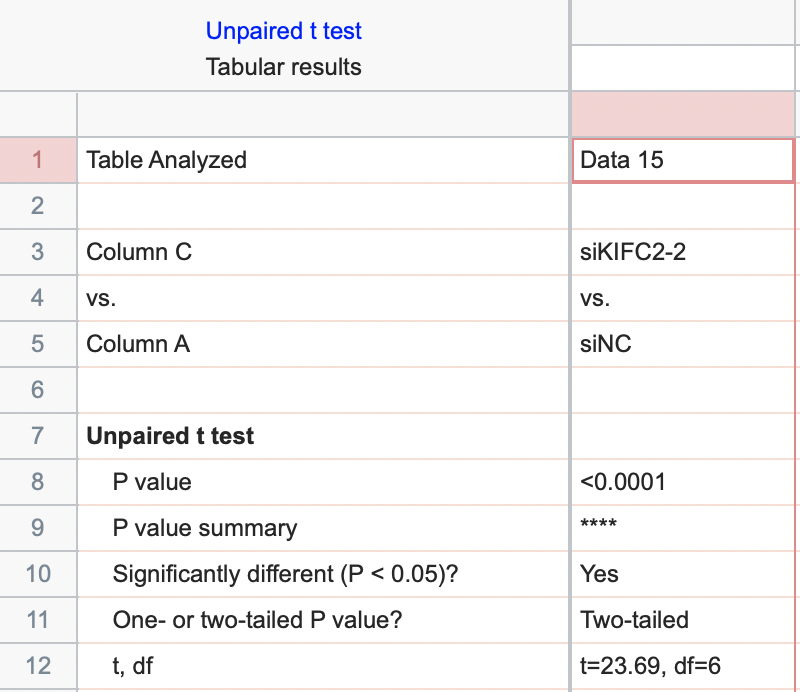


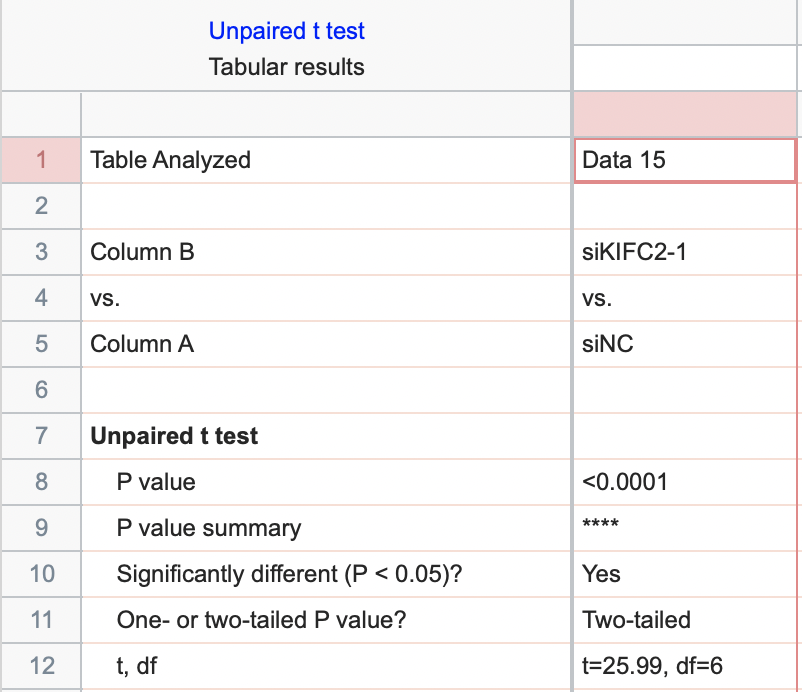

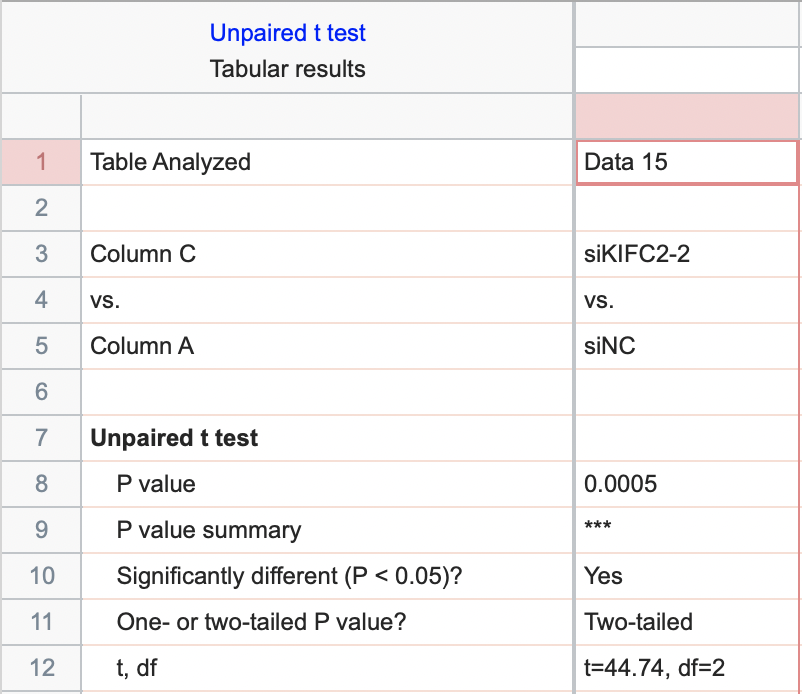


Figure 5G-PC3


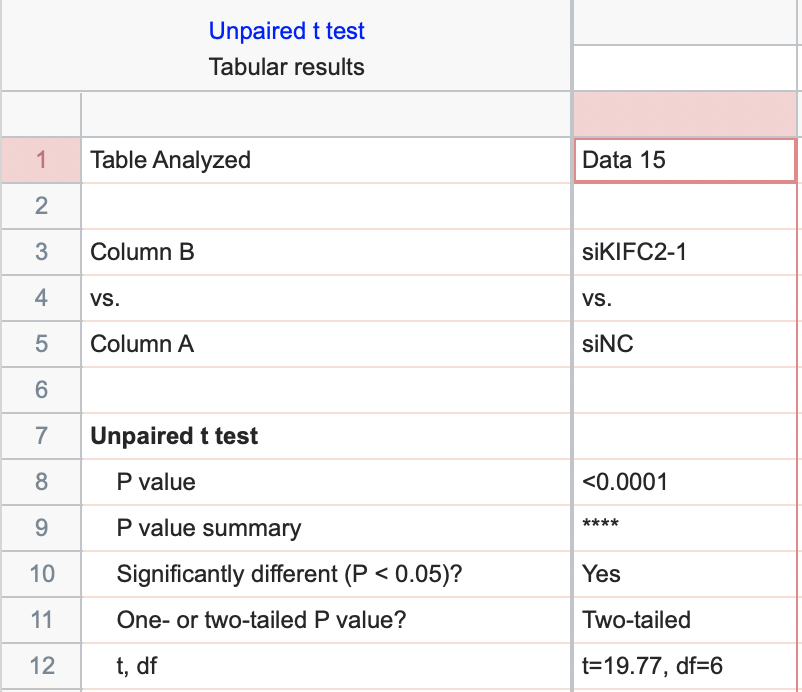

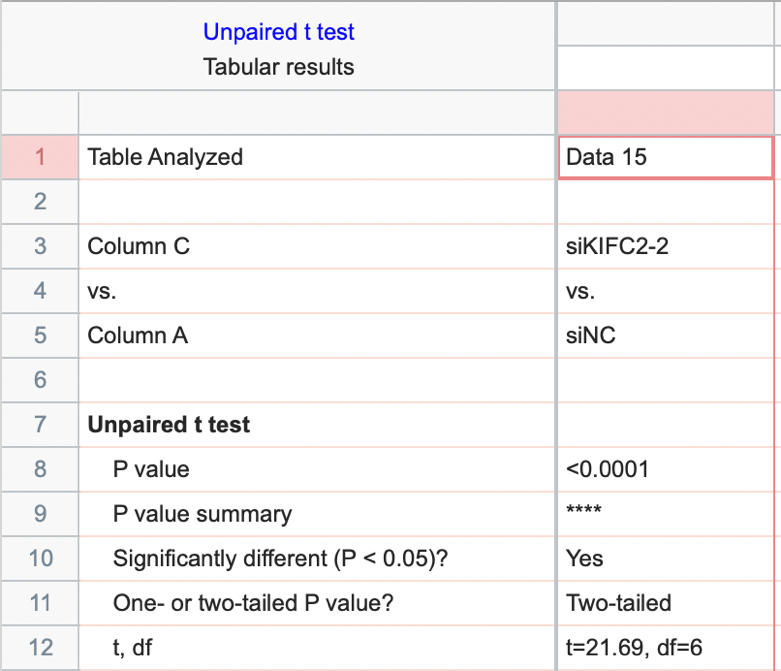


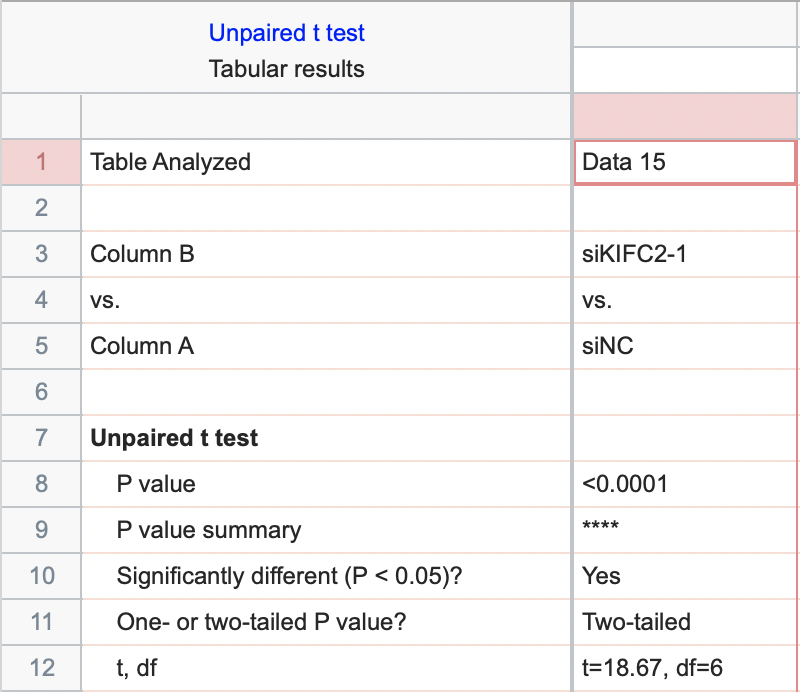

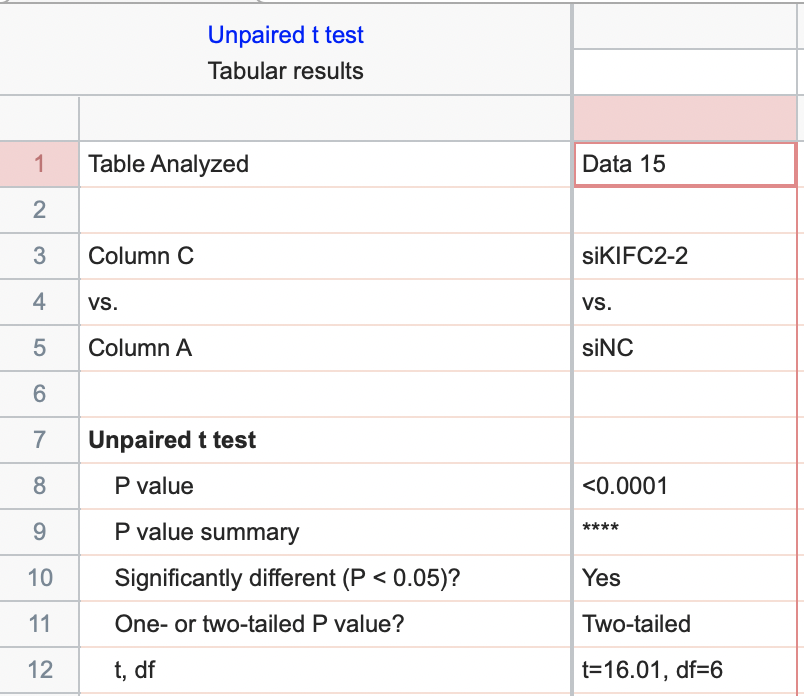


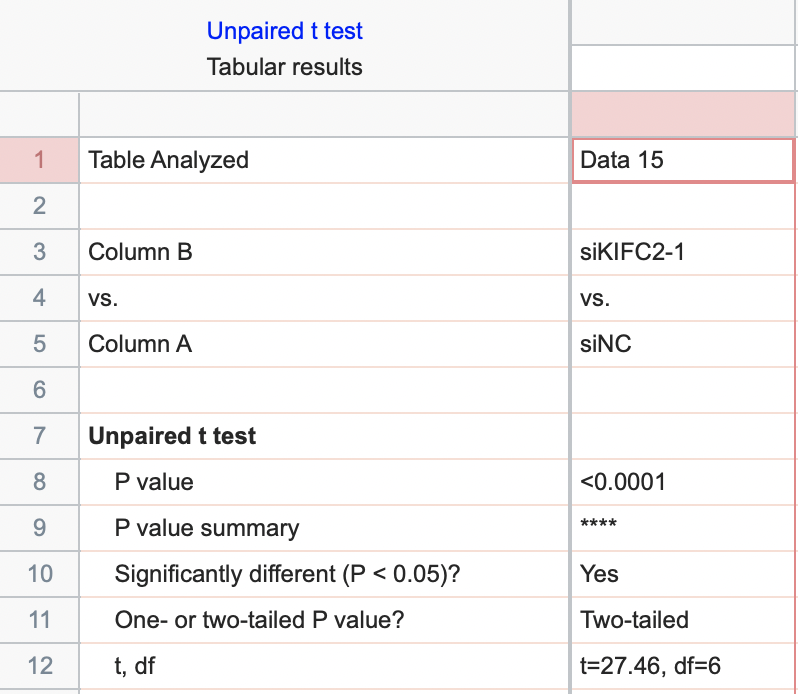

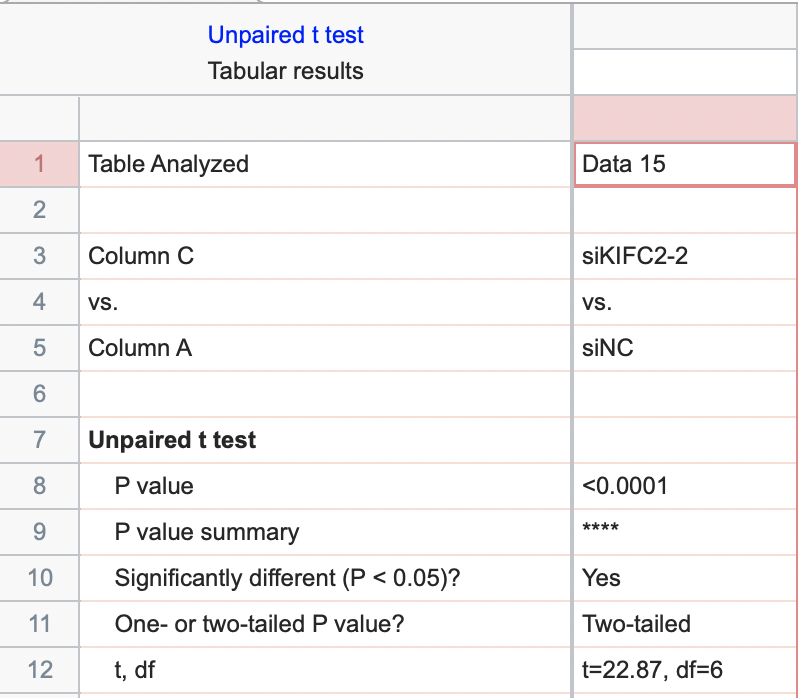


Figure 6A


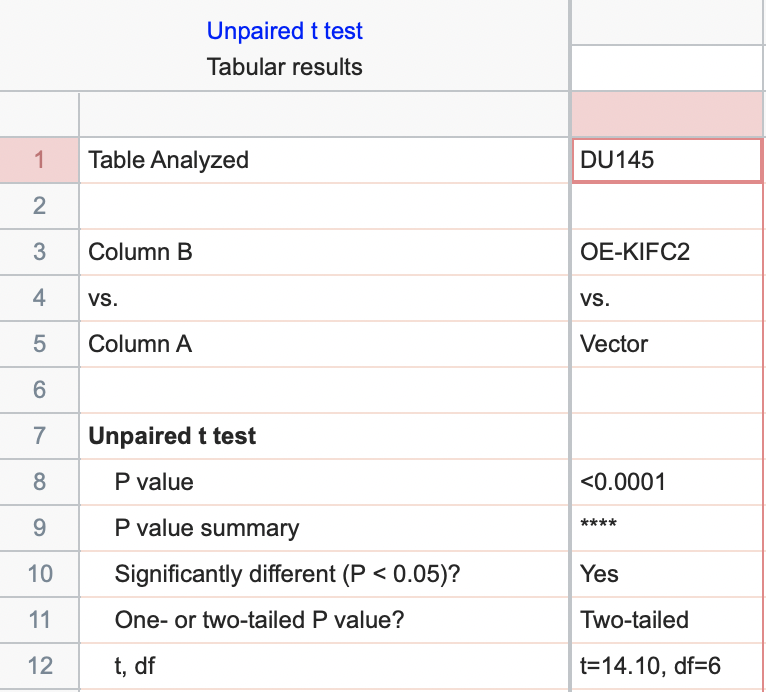

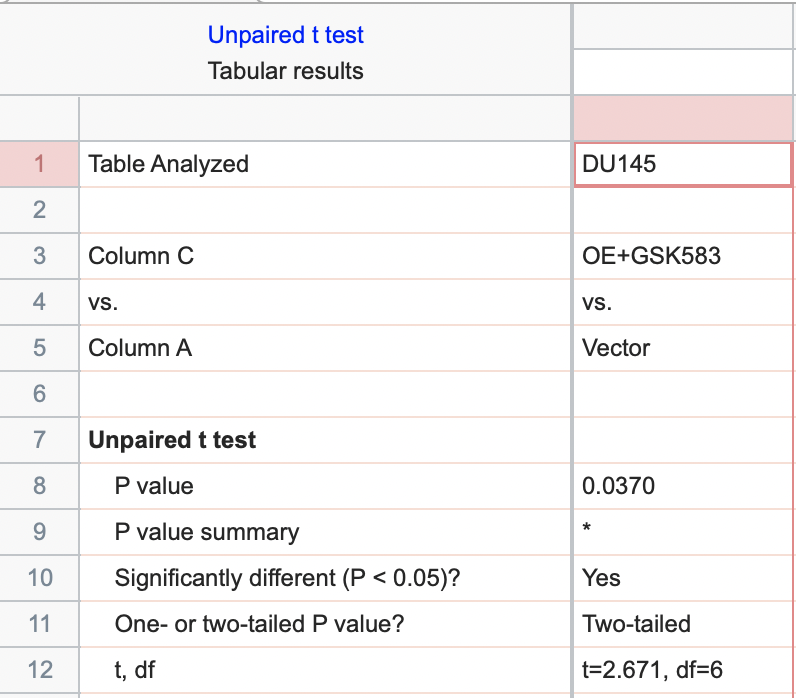


Figure 6B


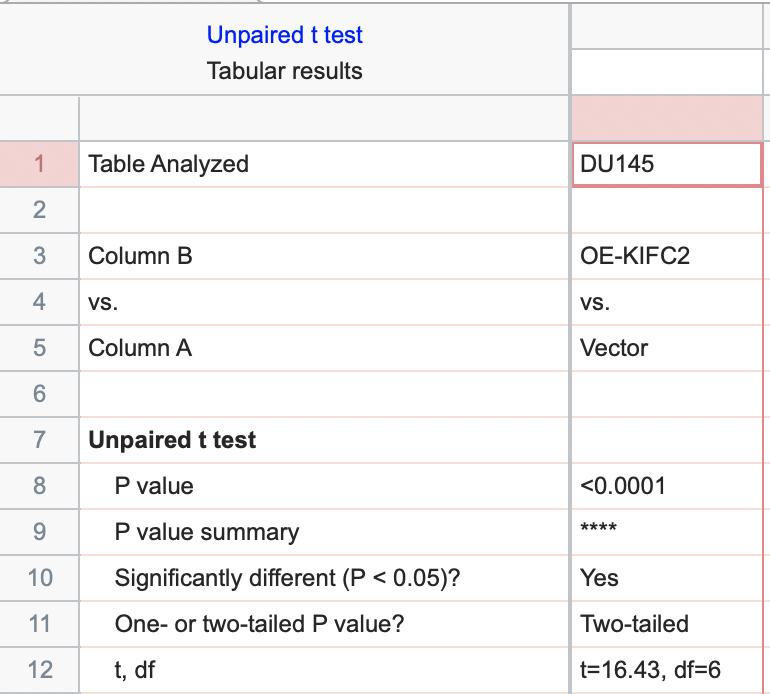

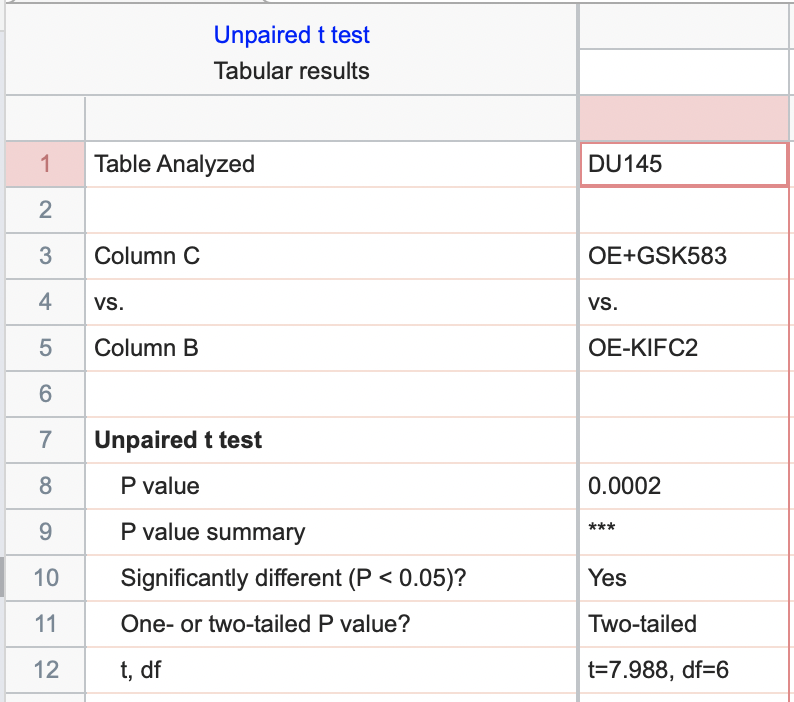


Figure 6E


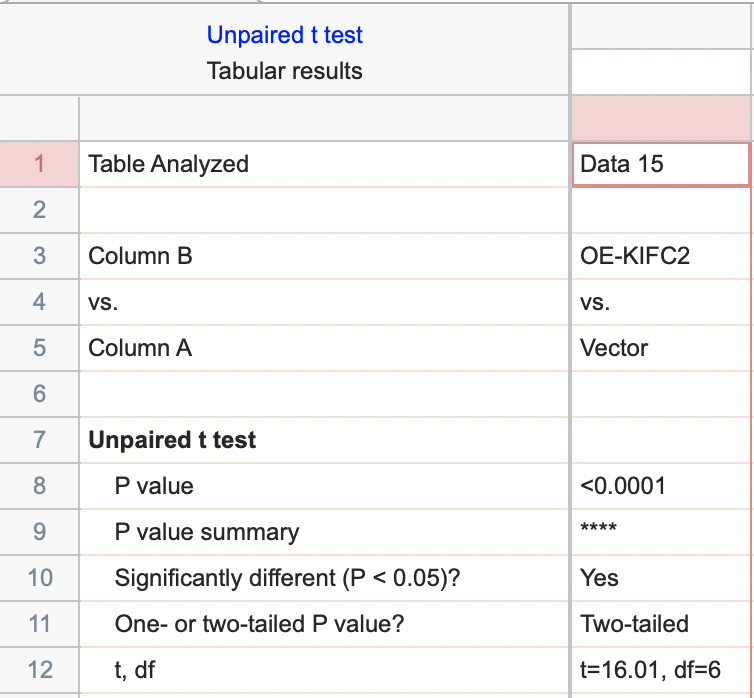

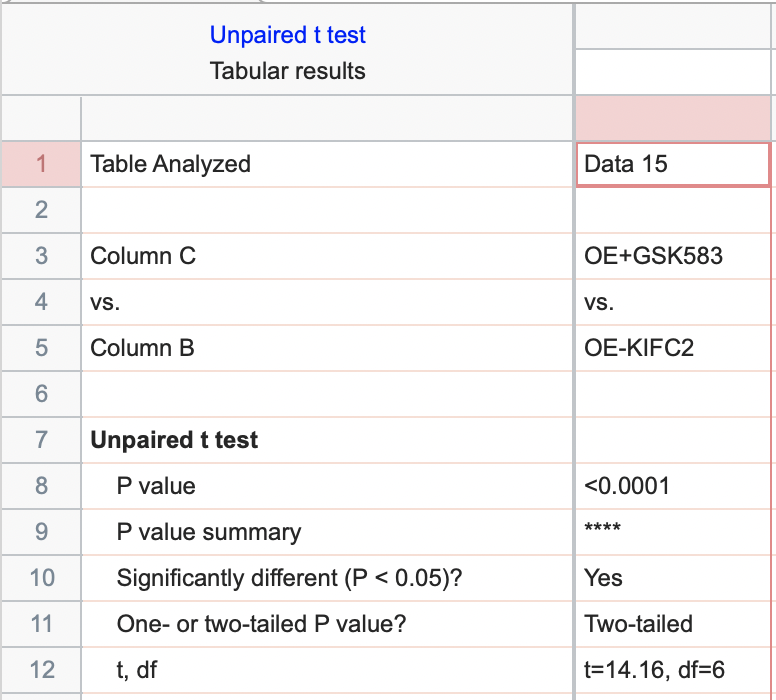


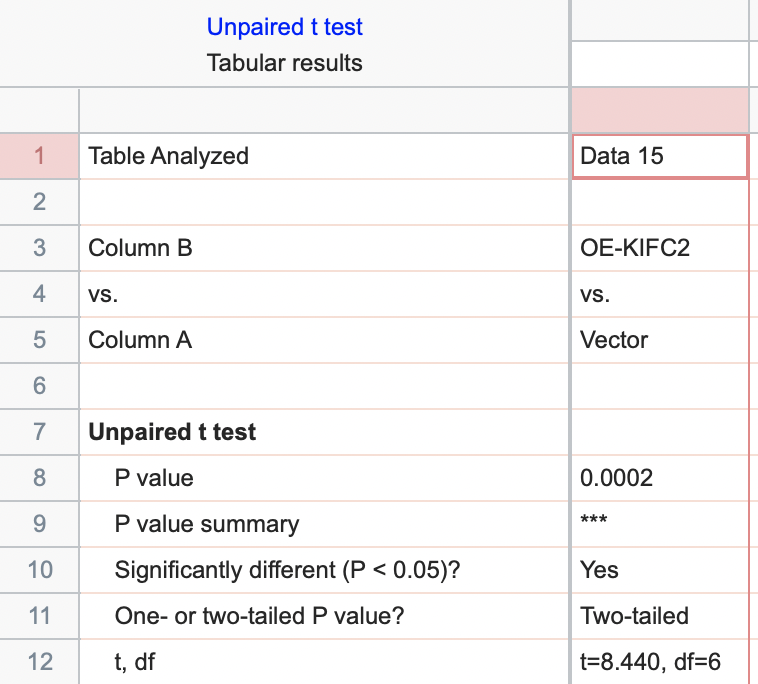

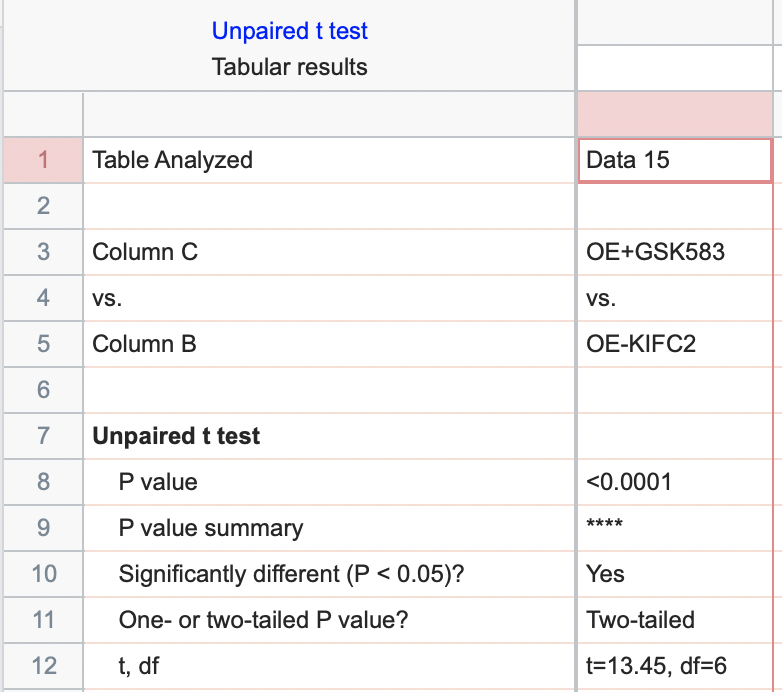

Supplement: Supporting information S1 [file mmc2.docx]
