# Supplementary figures and images for: The kinesin-14 family motor protein KIFC2 promotes prostate cancer progression by regulating p65
Source: J Biol Chem. 2023 Sep 14;299(11):105253. doi: 10.1016/j.jbc.2023.105253 (PMC10590982; doi:10.1016/j.jbc.2023.105253)

Figure 2G

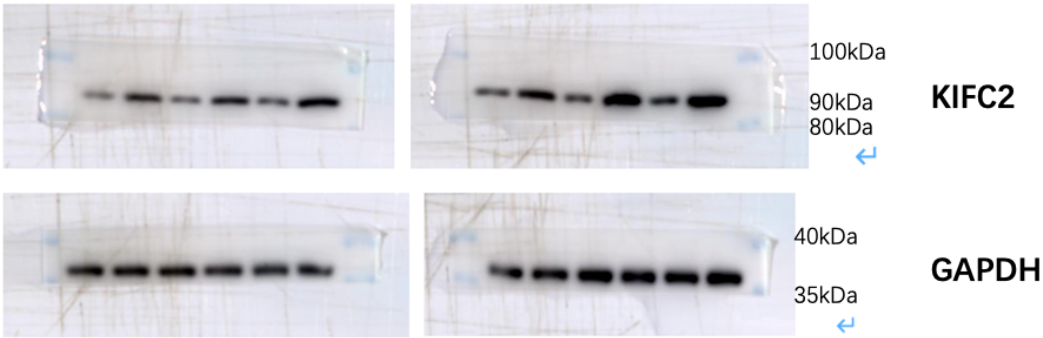

↩

↩

Figure 3A

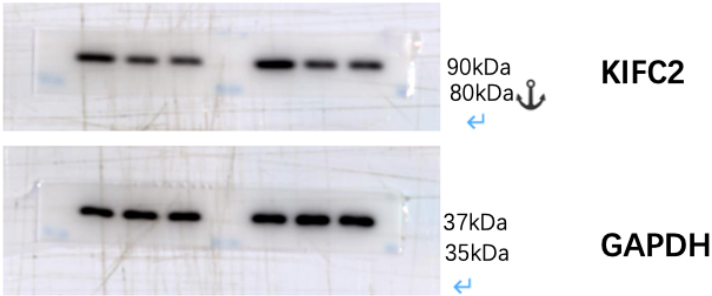

↩

↩

Figure 4A

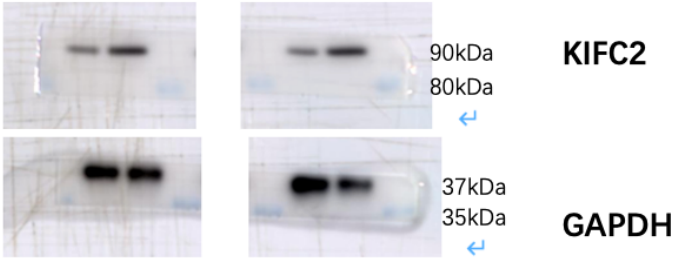

↩

Figure 5D

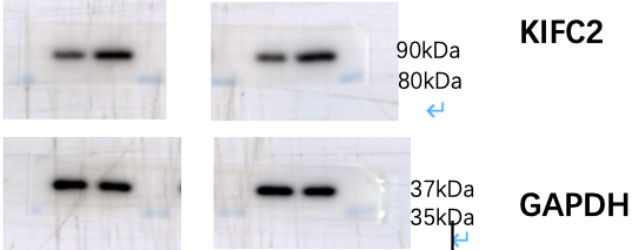

Figure 5E

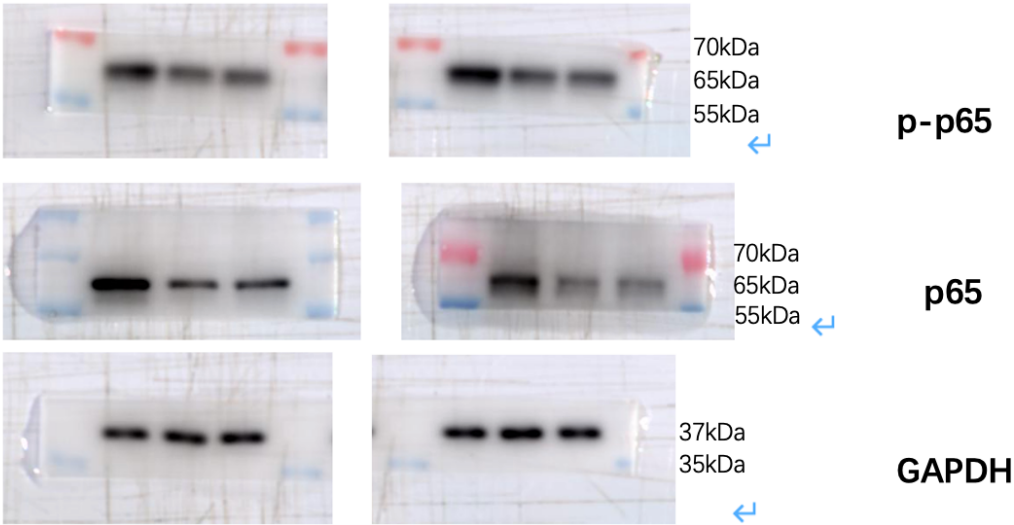

←

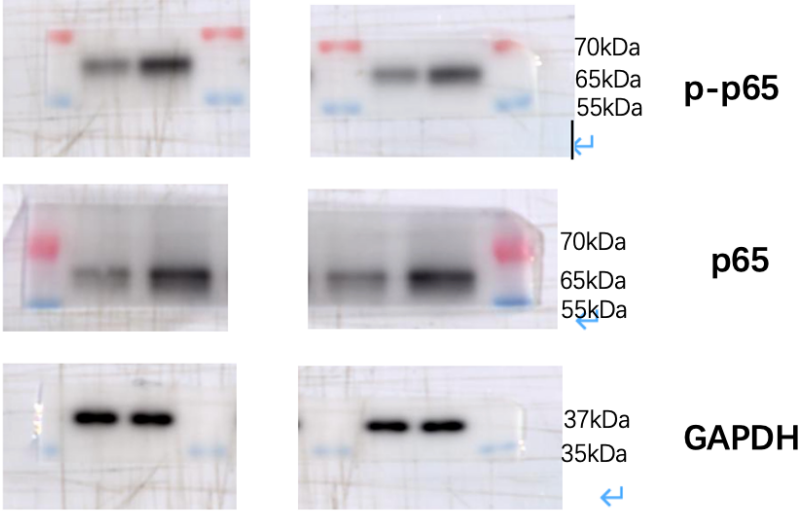

Figure 5|

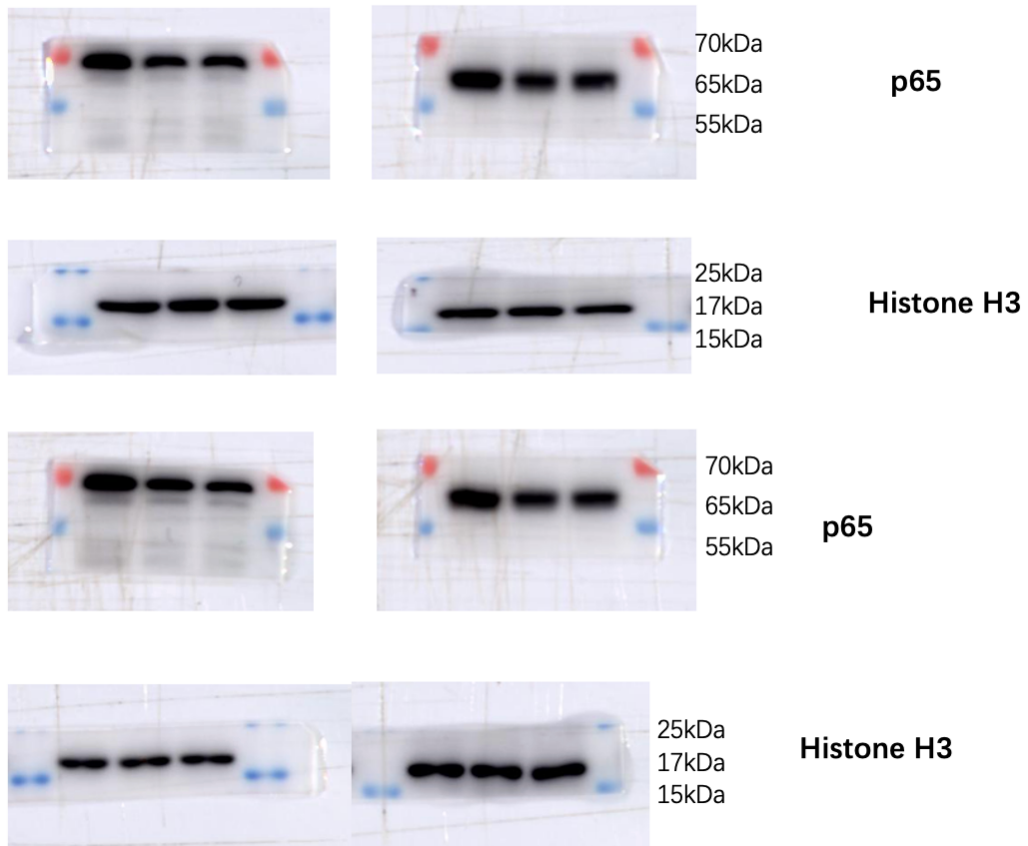

Supplement: Supporting information S2 [file mmc3.pdf]
